# *trans*-Symmetric Dynamic Covalent Systems: Connected Transamination and Transimination Reactions

**DOI:** 10.1002/chem.201500520

**Published:** 2015-06-03

**Authors:** Fredrik Schaufelberger, Lei Hu, Olof Ramström

**Affiliations:** [a] Department of Chemistry, KTH - Royal Institute of Technology Teknikringen 30, 10044 Stockholm (Sweden) E-mail: ramstrom@kth.se

**Keywords:** dynamic covalent chemistry, isomerization, Schiff bases, systems chemistry, transamination

## Abstract

The development of chemical transaminations as a new type of dynamic covalent reaction is described. The key 1,3-proton shift is under complete catalytic control and can be conducted orthogonally to, or simultaneous with, transimination in the presence of an amine to rapidly yield two-dimensional dynamic systems with a high degree of complexity evolution. The transamination–transimination systems are proven to be fully reversible, stable over several days, compatible with a range of functional groups, and highly tunable. Kinetic studies show transamination to be the rate-limiting reaction in the network. Furthermore, it was discovered that readily available quinuclidine is a highly potent catalyst for aldimine transaminations. This study demonstrates how connected dynamic reactions give rise to significantly larger systems than the unconnected counterparts, and shows how reversible isomerizations can be utilized as an effective diversity-generating element.

## Introduction

Constitutional dynamic chemistry (CDC) involves studies of chemical systems that respond to stimuli and adapt to external or internal pressure.[1] In this context, the systemic properties that emerge through interactions between components in a dynamic compound mixture can be very different from those of the isolated individual members. The systems are also fundamentally under thermodynamic control, in which reversible covalent bonds and noncovalent interactions allow access to the most energetically stable state, from which adaptation can occur. The area has expanded rapidly during recent years, and many applications for dynamic systems within ligand/receptor interaction studies, materials chemistry, catalysis, and molecular sensors have been developed.[2] Furthermore, CDC provides a relevant framework for the emerging field of systems chemistry.[3]

However, a challenge in CDC has been the relatively low number of suitable reversible covalent bonds that partake in dynamic systems. To generate large, diverse dynamic systems for different applications, it is of high importance to develop more reversible covalent connections capable of exchange under mild conditions. Only a few dynamic covalent-bond functionalities, mainly imine/acyl hydrazone[4] and disulfide exchange,[5] are typically used in dynamic systems, and despite recent developments of reversible transformations, such as dynamic thiol ester exchange,[6] the Strecker reaction,[7] nitrone exchange,[8] the nitroaldol reaction,[9] transamidation,[10] and alkyne metathesis,[11] there is still a growing demand for new reversible bonds that can be utilized in the construction of complex networks and systems.

Dynamic covalent bonds that can operate orthogonally to, or simultaneous with, other reversible functionalities are, in this respect, of particular interest. Compared with systems based on single dynamic reactions, such multidimensional arrays lead to significantly larger systems with higher diversity, potentially covering more chemical space. A range of examples of orthogonal covalent and noncovalent reactions, mostly based on metal coordination, acyl hydrazones, and disulfide chemistry, have also been reported.[7, 12]

In this context, we envisaged that the coupling of reversible isomerization to intermolecular dynamic exchange would provide rapid entry to highly complex dynamic systems. However, isomerization reactions constitute an underexplored area of CDC; the only example involves fluxional systems of bullvalene derivatives.[13]

**Scheme 1:**
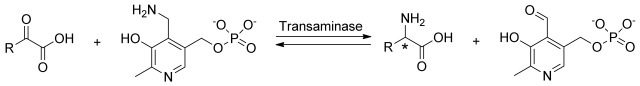
Reversible transamination of α-ketoacids under transaminase catalysis.

The reversibility of azomethine transamination is well known.[14] In biological systems, transaminases catalyze both transamination and transimination of amino acids to and from α-ketoacids with pyridoxal-5′-phosphate as a cofactor (Scheme 1). The transformation is of high industrial interest, and biocatalytic equilibrium control is also heavily pursued for the production of chiral amines.[15]

Renewed interest has also emerged in nonbiological systems, for which transamination strategies have been developed in, for example, asymmetric organocatalysis, synthesis of fluorinated amines, and selective N-terminal functionalization of peptides.[16] The synthetic challenge with transaminations resides in often unfavorable equilibria, with typical equilibrium constants near unity. From a CDC viewpoint, however, this challenge is instead an advantage, since it allows a predictable constituent expression in the systems.

Herein, we report the development of reversible transamination of aromatic imines and orthogonal coupling to transimination, which yields double dynamic imine systems. These transamination–transimination (TATI) systems exhibit interesting properties because each of the two individual dynamic reactions can be toggled on or off by the addition or removal of the respective catalyst for each process. Since both dynamic reactions manipulate the same functional group, combining the two dynamic exchange processes leads to connected dynamic systems with unusually large complexity evolution.

## Results and Discussion

### Theoretical analysis of dynamic exchange

**Figure 1:**
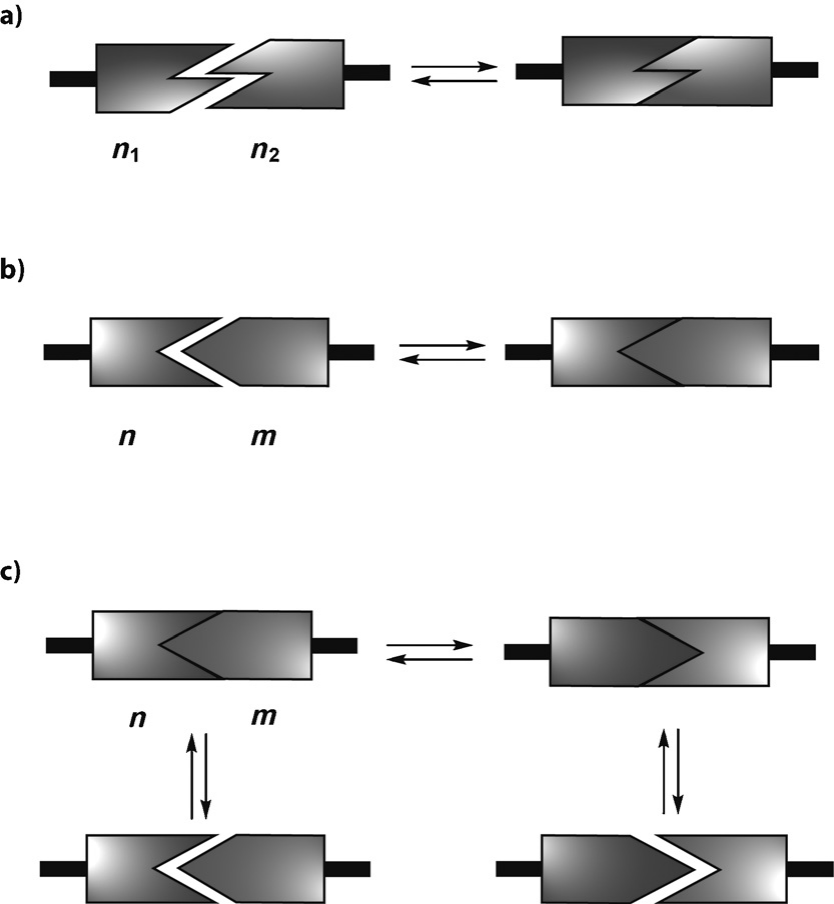
Illustration of the main classes of dynamic bonds in CDC. a) Symmetric dynamic exchange; b) unsymmetric dynamic exchange; c) *trans*-symmetric dynamic exchange.

Figure 1 a and b illustrates the two main types of dynamic covalent bonds used in CDC. For symmetric dynamic bonds, both exchange partners belong to the same class of functional groups, of which disulfide exchange and alkene metathesis are two prominent examples.1

For such a system, the number (*N*) of generated system constituents upon addition of *n* different types of initial monofunctional components is given by Equation (1):


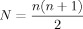
(1)

For unsymmetric exchange, the two partners belong to different functional groups, such as aldehydes and amines in imine exchange chemistry. With *n* monofunctional components of one class and *m* of the other, the number (*N*) of constituents becomes that given by Equation (2):


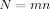
(2)

In this study, conditions to dynamically alter the nature of the components of an unsymmetric reversible connection have been developed, so that the two functional groups interconvert into each other (Figure 1 c). This scenario, which we term *trans*-symmetric exchange, will in most instances give rise to a number (*N*) of system constituents predicted by Equation (3):[17]


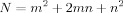
(3)

From inspection of Equations (1)–(3), it is clear that a TATI system will generate systems that are at least (for *n*=*m*) four times larger than the analogous unsymmetric imine system. Thus, the diversity of screening collections generated by *trans*-symmetric exchange will be considerably higher than that for other exchange types.

### Transamination catalysis

Imine **1** was initially investigated in an isolated transamination reaction (Table 1), in which many of the previously reported conditions proved unsuitable. For example, the use of strong heating or strong bases, such as *t*BuOK and 1,5-diazabicyclo[5.4.0]undec-5-ene (DBU), instigated rapid decomposition, which generated complex mixtures well before the transamination equilibrium could be reached. Finally, successful clean transamination could be achieved by using the conditions developed by Soloshonok and co-workers, with a large excess of NEt_3_ in MeCN at 50 °C.[18] After 24 h, about 66 % of imine **1** had undergone transamination to form isomer **2**. Extending the reaction time to 30 h led to arrival at the equilibrium point, with a product distribution of 25/75 in favor of transamination product **2**. This result is in agreement with theory because the equilibrium position of benzylic aldimine/aldimine transaminations can be correlated with the Hammett substituent parameters; more electron-rich systems are favored to exhibit the imine functionality.[14]

**Table 1:** Initial optimization of the transamination reaction^[a]^

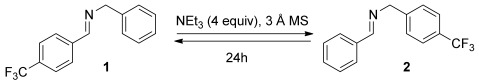			
Solvent	*T* [°C]	Distribution1/2^[b]^	Equilibrium1/2
MeCN	20	no reaction	n.d.^[c]^
MeCN	50	34/66	25/75
CHCl_3_	50	93/7	n.d.^[c]^
toluene	50	94/6	n.d.^[c]^
THF	50	79/21	n.d.^[c]^
DMF	50	30/70	27/73
DMSO	50	34/66	26/74

[a] Conditions: imine **1** (0.25 mmol), NEt_3_ (1.0 mmol), 3 Å molecular sieves (MS; 10 mg), anhydrous solvent (0.25 mL), 24 h, N_2_. [b] Analyzed by ^1^H NMR spectroscopy; [c] n.d.=not determined.

A reversible covalent reaction for CDC needs to display long-term stability, mild conditions, and rapid kinetics. Thus, the use of a large excess of NEt_3_, along with the relatively long equilibrium time, highlighted the need for an improved protocol. The initial screening (Table 1) indicated that the highest rates occurred in the presence of NEt_3_ in polar aprotic solvents; anhydrous MeCN, DMSO, and DMF provided access to equilibrium compositions in slightly longer than 24 h. Since the use of the last two solvents led to complications in product isolation and reaction monitoring, MeCN was adopted in the next optimization step.

The use of less NEt_3_ resulted in significant retardation of the equilibration rate (Table 2, entry 2). To be able to decrease the catalyst loading, a more suitable Brønsted base was thus deemed necessary.2

**Table 2:** Base optimization studies for reversible transamination^[a]^

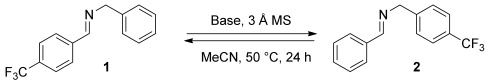				
Entry	Base	Equivalents	Distribution1/2^[b]^	Equilibrium conversion [%]^[c]^
1	NEt_3_	4	34/66	88
2	NEt_3_	1	67/33	44
3	NMI	4	no reaction	–
4	NMM	4	93/7	9
5	NMP	4	92/8	11
6	DIPEA	4	30/70	93
7	DMAP	4^[d]^	71/29	39
8	DABCO	4	25/75	100
9	**C1**	4	25/75	100
10	**C2**	4	26/74	100
11	DABCO	0.2	71/29	39
12	**C2**	0.2	43/57	76
13	**C1**	0.2	25/75	100
14	**C3**	0.2^[d]^	98/2	3
15	**C4**	0.2	98/2	3
16	**C5**	0.2	no reaction	–
17	**C1**	0.1	35/65	87
18	**C1**	0.05	50/50	66
19	DBU	0.1	decomposition	–
20	TMG	0.1	decomposition	–

**Figure 2:**
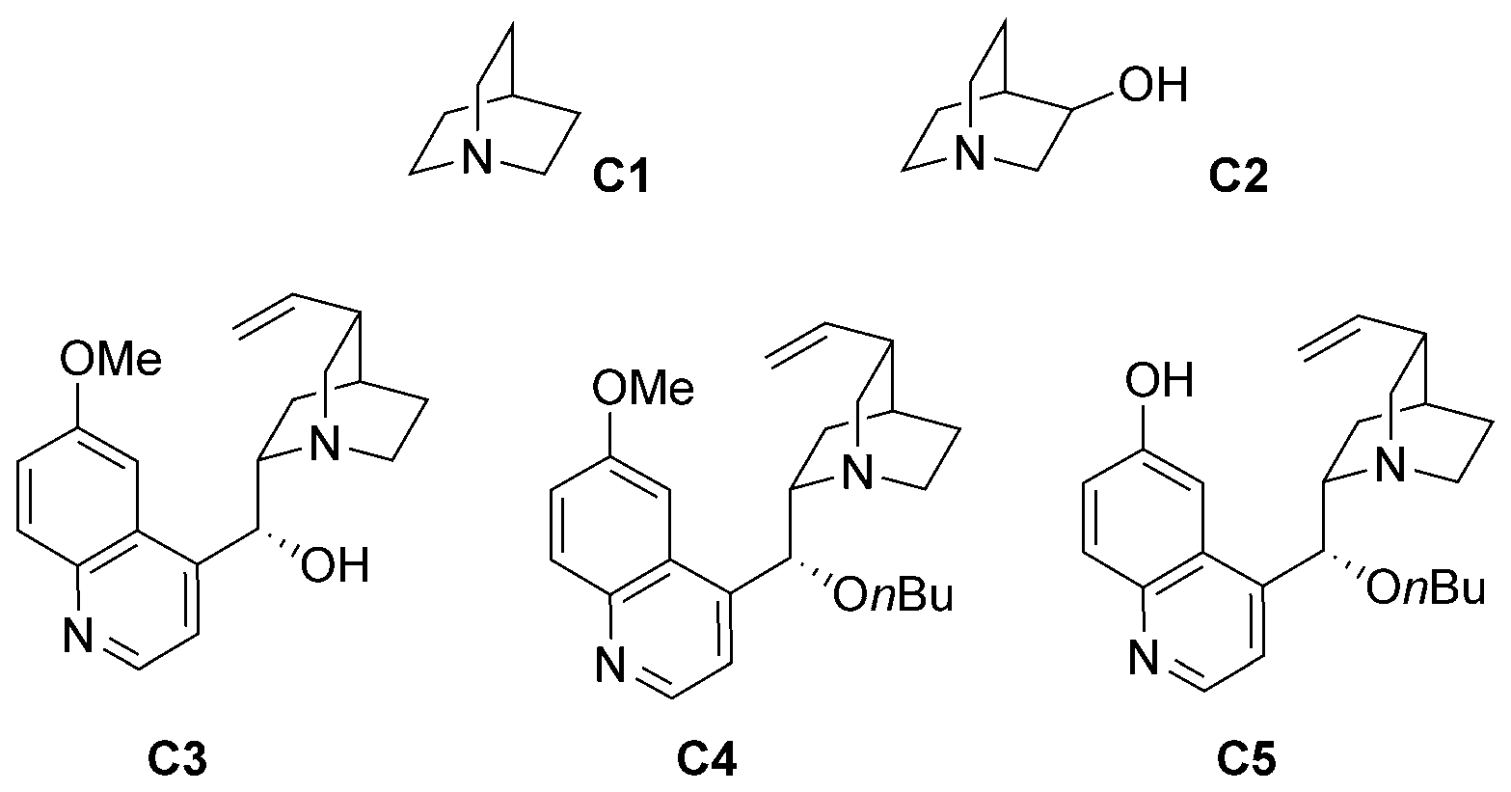
Quinuclidine-based catalysts evaluated for transamination activity.

[a] Conditions: imine **1** (0.25 mmol), base (*n* equivalents), 3 Å MS (10 mg), anhydrous MeCN (0.25 mL), 50 °C, N_2_, 24 h. NMI=*N*-methylimidazole, NMM=*N*-methylmorpholine, NMP=*N*-methylpyrrolidine, DIPEA=diisopropylethylamine, DMAP=4-dimethylaminopyridine, DABCO=1,4-diazabicyclo[2.2.2]octane, TMG=*N*,*N*,*N*′,*N*′-tetramethyl-1,3-propanediamine; for the structures of **C1**–**C5** (see Figure 2). [b] Analyzed by ^1^H NMR spectroscopy. [c] Conversion towards the equilibrium position, 25/75 for **1**/**2**. [d] Low solubility.

**Scheme 2:**
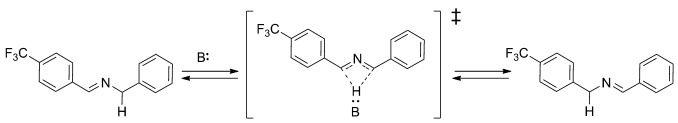
Proposed concerted proton shuffling during the chemical transamination of aldimines.[18]

Mechanistic studies have indicated that many base-catalyzed 1,3-proton shifts may proceed through a concerted proton shuffling mechanism (Scheme 2).[18] The transamination rate should therefore not only be dependent on the base strength, but also on steric congestion around the basic site. A more accessible nitrogen center should be able to provide more efficient proton shuffling.

Thus, a range of bases with less steric bias were screened. As expected, the position of the equilibrium did not change significantly upon variation of base or base loading. Weak bases, such as NMI and NMM, showed low activity (Table 2, entries 3 and 4), as did the stronger base NMP, which somewhat surprisingly provided only a modest equilibration rate (Table 2, entry 5). However, the use of 4 equivalents of quinuclidine **C1**, 3-hydroxyquinuclidine **C2**, or DABCO led to efficient transamination, reaching equilibria within 24 h. Drastically lowering the loadings of these three catalysts to 0.2 equivalents still led to decent rates, with **C1** providing the best performance with an equilibrium reached in around 20 h. As little as 5 mol % **C1** could be utilized for the transformation, albeit at the cost of equilibration time.

Due to their successful application in the asymmetric transamination of α-ketoesters and the high similarity of the active basic site to that of **C1**, cinchona alkaloids were also evaluated as catalysts (Figure 2).[16b] However, quinine **C3** was almost completely inactive, possibly due to low solubility (Table 2, entry 14). Alkylation of the hydroxyl functionality yielded soluble catalyst **C4**, but no increase in activity could be observed (Table 2, entry 15). Compound **C5** was furthermore evaluated, since demethylation of the quinoline methoxy group could reveal a stabilizing hydrogen-bond-donor functionality, but this was also found to be completely inactive (Table 2, entry 16). Because cinchona alkaloids are significantly bulkier around the quinuclidine nitrogen than **C1**, these results again indicate that the transamination reaction is strongly dependent on the steric environment around the basic site.2

Because system stability is of utmost importance in dynamic chemistry applications, it was gratifying to observe that **C1** catalysis did not induce any degradation, even six days after equilibrium had been reached. Given the general sluggishness of aldimine transamination, catalyst **C1** seems to be a remarkably effective and mild catalyst, even from a synthetic perspective.

### Connected TATI

With conditions for the reversible transamination reaction at hand, transimination was next investigated.[19] A range of Lewis acids were evaluated as catalysts (see the Supporting Information), with most catalysts providing complete transimination of all tested substrates within 10 min. The most robust transimination conditions were achieved with ZnBr_2_,[7] although transimination also worked well with primary amine catalysis. The time to reach equilibrium did, however, increase to about 1 h in this case, compared with a few minutes for ZnBr_2_.

**Figure 3:**
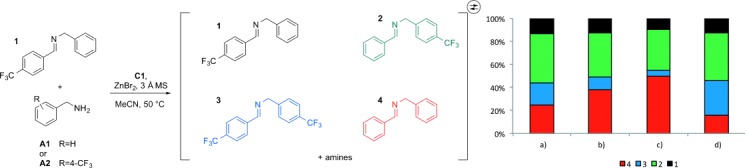
TATI equilibrium perturbation experiments. The relative system composition was dependent on the amine added: a) 0.1 equivalents of amine A1, b) 0.5 equivalents of amine A1, c) 1.0 equivalents of amine A1, d) 0.5 equivalents of amine A2. The system composition was analyzed by both ^1^H NMR spectroscopy and GC. Conditions: imine 1 (0.25 mmol), amine, C1 (0.05 mmol), ZnBr_2_ (0.0125 mmol), 3 Å MS (10 mg), MeCN (0.25 mL).

Connected TATI systems could be generated simply through the addition of both catalysts to the same mixture, as shown in Figure 3. Mixing imine **1** with benzylamine **A1**, ZnBr_2_, and base **C1** in MeCN, in the presence of 3 Å MS, led to the generation of four imines, as evidenced by both ^1^H NMR spectroscopy and GC/FID analysis. After around 30 h, no further system composition changes could be observed.

The system distribution at equilibrium could be easily tuned through amine addition. The use of 0.1 equivalents of benzylamine **A1** yielded a system of constituents **1**–**4** (Figure 3 a). Here, compound **2** constituted nearly 43 % of the total imine content, which indicated a preferred systemic expression of **2** over the other constituents under these conditions. In general, the system indicated a preference for releasing the less-basic free amine **A2** and incorporating higher amounts of more basic amine **A1** into the imine system. Increasing the amount of **A1** to 0.5 and 1.0 equivalents led to a clear, gradual increase in the proportion of imine **4** up to 50 % of the total imine content (Figure 3 b and c). However, by omitting amine **A1** and instead adding 0.5 equivalents of the trifluoromethyl-substituted benzylamine **A2**, the relative amount of compound **4** was drastically reduced and the expression of the doubly trifluoromethyl-substituted imine **3** instead increased (Figure 3 d). Curiously, the ratios of compounds **1** and **2** were relatively stable during these system manipulations, only varying slightly despite drastic changes in the overall system composition. This indicates that homosubstituted imines **3** and **4** are acting as “sinks” that modulate and buffer the concentration of imines **1** and **2** by preferential incorporation of newly added amine.

**Figure 4:**
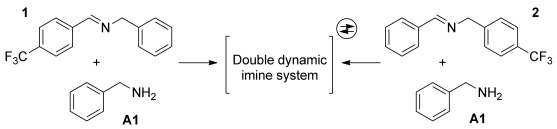
Results obtained from dual-entry-point equilibration analysis. The double dynamic systems were identical when generated from either direction. See the Supporting Information for further details and more control experiments.

The high level of adaptability strongly indicates that the connected system was under thermodynamic control. However, further support that the TATI system had reached equilibrium was provided by dual-entry-point analysis. Two identical systems were thus generated, starting from either compound **1** or **2**, as displayed in Figure 4. Upon addition of 0.5 equivalents of amine **A1** to a solution of imine in MeCN with **C1** and ZnBr_2_ as catalysts, both systems resulted in identical distributions, which remained stable over several days.4

### Kinetic studies

**Figure 5:**
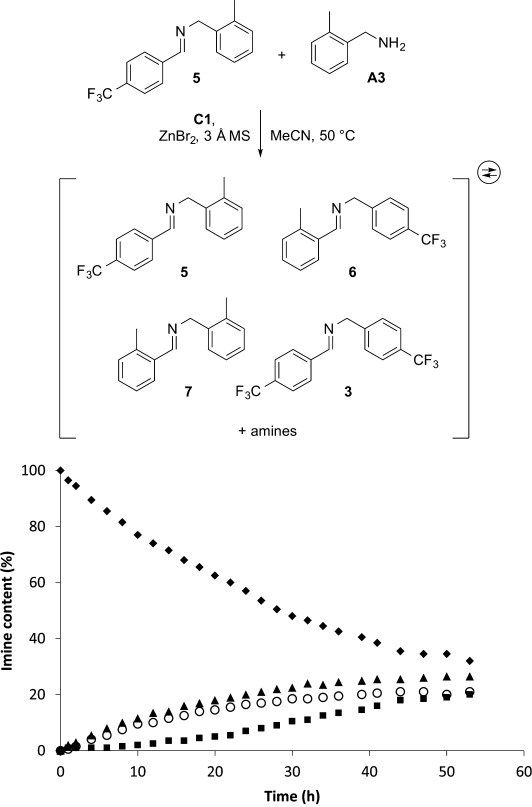
Kinetic profile for the four different imines in the TATI system (5 (⧫); 6 (▪); 7 (▴); 3 (○)), as measured by ^1^H NMR spectroscopy. Conditions: imine 1 (0.25 mmol), amine A3 (0.025 mmol), catalyst C1 (0.05 mmol), ZnBr_2_ (0.0125 mmol), 3 Å MS (10 mg), MeCN (0.25 mL). Data were obtained from duplicate experiments.

The kinetic profile of the dynamic system generation was next evaluated (Figure 5). Since the system based on imines **1**–**4** required GC analysis for full quantification due to overlapping signals in the NMR spectra, compound **5** and benzylamine **A3** were instead subjected to the TATI conditions. Careful reaction monitoring by NMR spectroscopy during the equilibration process revealed that transamination of compound **5** was the limiting step of the reaction system, in which equilibrium was reached after around 48 h. The concentration profile of compound **5** indicated an exponential decrease, which was symptomatic of first-order behavior with respect to the reagent. Immediately upon transamination, the new heterosubstituted imine **6** was formed. Transimination with amine **A3** immediately occurred to form compound **7**, releasing benzylamine **A2**, which, in turn, underwent transimination with starting compound **5** to yield the second homosubstituted imine **3**. Interestingly, the concentration profile of direct transamination product **6** turned out to be almost sigmoidal in shape. During a long induction period, the system seemed to settle into local transimination equilibria, in which compound **6** was an under-expressed constituent. Only once a certain ratio of free amines **A2** and **A3** had been formed did compound **6** start to accumulate, eventually reaching approximately the same concentration as imine **3**. NMR spectroscopy analysis confirmed that the free amine ratio stayed relatively constant after 18 h, which again provided support for a model in which the homosubstituted imines acted as amine sinks during equilibration. This unexpected effect is an example of the intricate kinetic behavior of interconnected chemical systems and highlights the need for dynamic reaction networks to be analyzed from a broader systems chemistry perspective.

### Dynamic pathway control

**Figure 6:**
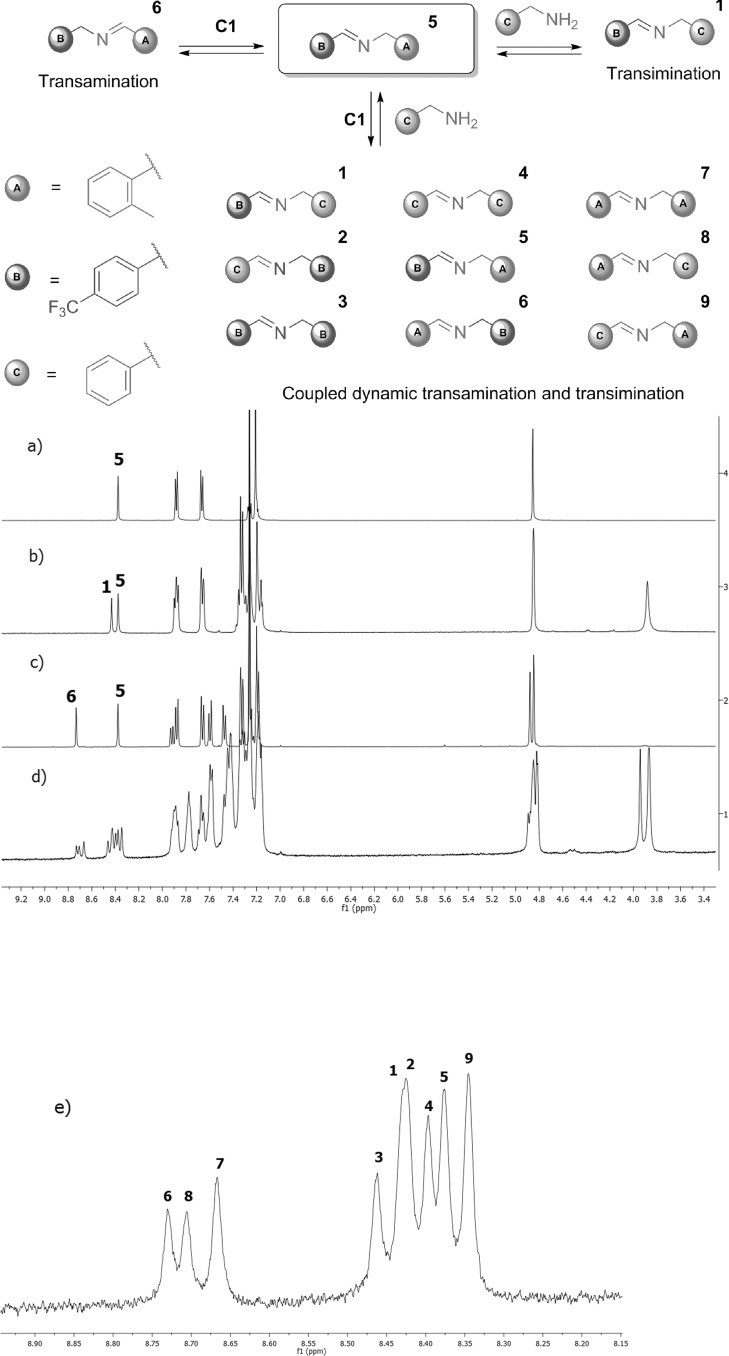
^1^H NMR spectra illustrating selective access to each equilibration mode of imine 5: a) starting material 5; b) transimination equilibrium (15 min) with imine 5 (1.0 equiv), benzylamine A1 (1.0 equiv), ZnBr_2_ (0.05 equiv); c) transamination equilibrium (24 h) between imines 5 and 6, with C1 (0.2 equiv); d) coupled transamination and transimination equilibrium (45 h) with A1 (1.0 equiv), 5 (1.0 equiv), C2 (0.2 equiv), and ZnBr_2_ (0.05 equiv). e) Enlarged view of the characteristic imine resonance region. Resonances corresponding to compounds 1 and 2 overlap.

The individual transimination and transamination equilibria of model compound **5** were easily accessible by selection of the reagent, and represent two orthogonal reversible reaction pathways in the dynamic covalent reaction network (Figure 6). However, the key diversity generation originates from a combination of the two pathways. When mixing compound **5** with transamination catalyst **C1** and benzylamine **A1** (1 equiv), a system of nine imine compounds and three amines was efficiently generated (Figure 6). This experiment verifies the *trans*-symmetric exchange mode of the system and illustrates the potential in the TATI protocol for rapid systemic complexity generation. The system size growth relative to the amount of starting compounds is thus demonstrated to be significantly higher than that in non-*trans*-symmetrical protocols utilizing monofunctional exchange partners.6

### Substrate scope and synthetic considerations

Finally, the substrate scope of the TATI system formation was investigated in detail. As displayed in Table 3, a range of substrates were compatible with the optimized reaction conditions. Generally, the system needed at least one electron- withdrawing component on one of the aromatic rings for acceptable equilibration times. This can be understood when considering the reaction mechanism, in which the initial transamination proton shuffling is the rate-limiting step. Because electron-withdrawing groups on the aromatic rings increase the acidity of the benzylic protons, more electron-poor systems should lead to faster transamination.3

**Table 3:** Substrate scope of TATI equilibration^[a]^

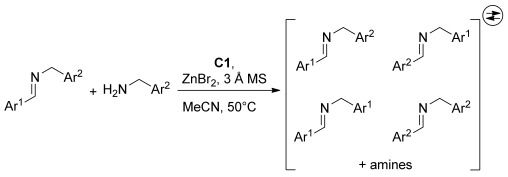					
Entry	Imine	Ar^1^	Ar^2^	*t* [h]^[b]^	Comment
1	**1**	4-CF_3_-C_6_H_4_	Ph	30	–
2	**10**	3-CN-C_6_H_4_	Ph	30	–
3	**11**	4-Br-C_6_H_4_	Ph	80	–
4	**12**	2-OH-C_6_H_4_	Ph	24	–
5	**13**	2,4-Cl-C_6_H_3_	Ph	48	–
6	**14**	4-NO_2_-C_6_H_4_	Ph	–	rapid decomposition
7	**14**	4-NO_2_-C_6_H_4_^[c]^	Ph	12	degrades within 48 h
8	**15**	4-pyridyl^[d]^	Ph	16	–
9	**16**	2-pyridyl^[d]^	Ph	16	–
10	**17**	1-*N*-Me-imidazoyl	Ph	24	–
11	**18**	2-furyl^[e]^	Ph	96	degradation during equilibration
12	**19**	2-MeO-C_6_H_4_	Ph	–	very slow
13	**20**	4-Me-C_6_H_4_	Ph	–	no reaction
14	**21**	4-F-C_6_H_4_	Ph	–	no reaction
15	**22**	2-naphthyl^[e]^	Ph	168	degradation during equilibration
16	**23**	4-CF_3_-C_6_H_4_	4-F-C_6_H_4_	36	–
17	**24**	4-CF_3_-C_6_H_4_	4-MeO-C_6_H_4_	42	–
18	**25**	4-CF_3_-C_6_H_4_	3,4,5-MeO-C_6_H_2_	42	–
19	**5**	4-CF_3_-C_6_H_4_	2-Me-C_6_H_4_	48	–

[a] Conditions: imine (0.25 mmol), amine (0.125 mmol), **C1** (0.05 mmol), ZnBr_2_ (0.0125 mmol), 3 Å MS (10 mg), MeCN (0.25 mL). [b] Approximate time for the system to reach equilibrium. [c] At RT with four equivalents of NEt_3_ instead of compound **C1**. [d] With 0.1 equivalents of amine, no ZnBr_2_. [e] With 0.5 equivalents of compound **C1**.

Cyano, trifluoromethyl, chloro, and bromo substituents all worked with the system, and different *ortho*-, *meta*-, and *para*-substituted compounds were all well tolerated. The strongly electron-withdrawing nitro-substituted imine **14** decomposed under the reaction conditions (Table 3, entry 6). Problematic behavior of nitro-substituted compounds in aldimine transaminations has been observed before.[14] Reverting to the use of less reactive NEt_3_ at room temperature led to quick equilibration, although the system decomposed over the next 48 h (Table 3, entry 7). Heteroaromatic systems generally performed well, albeit without the addition of ZnBr_2_ and with a lower amount of free amine with pyridine-based structures due to complexation and degradation of the picolylamines formed (Table 3, entries 8 and 9). With some compounds, such as imidazole imine **17** and furyl imine **18**, small amounts of unidentified side products appeared during equilibration.

Generally, compounds with only electron-donating substituents underwent very slow transamination (Table 3, entries 12–14). An exception was imine **12**, which reacted smoothly to provide a full TATI system in a short time (Table 3, entry 4). One reason for this reactivity could be intramolecular hydrogen bonding between the phenolic proton and the imine nitrogen.

Furthermore, benzylic imines with substituents on both aromatic rings were compatible with the system (Table 3, entries 16–19). Even methyl- and methoxy-substituted aromatics participated readily in the exchange processes, as long as at least one other component had a sufficiently electron-withdrawing functional group attached. The high compatibility range of these model substrates indicates that TATI exchange can be utilized with a range of substituted aromatic and heteroaromatic exchange partners, covering a wide part of the relevant chemical space in terms of aromatic compounds.

**Scheme 3:**
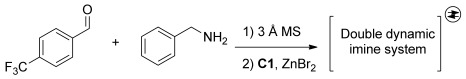
One-pot protocol for the direct creation of TATI dynamic systems from aldehydes and amines.

An advantage of TATI systems from a practical standpoint is the dual nature of the involved functional groups. If a particularly interesting aldehyde or amine is commercially unavailable or difficult to access synthetically, the reverse compound can instead be employed and the desired compound created for the screening collection in situ. This is, for example, the case for entries 10 and 15 in Table 3, for which very expensive benzylamines are created in situ from cheap, widely available aldehydes. It is also straightforward to form the benzylic imine in the flask before equilibration to generate a one-pot protocol that omits the extra imine formation step. Simply condensing the aldehyde and benzylamine in the presence of 3 Å MS, as shown in Scheme 3, followed by addition of the catalysts, led to the evolution of an identical system to that observed with preformed imine **1**.

Furthermore, simple aqueous washing led to the removal of all catalysts and benzylamines, which allowed the isolation of imine mixtures in high purity and yield. The imines can also be readily hydrolyzed, leading to aldehyde mixtures. This protocol could thus in theory also be of use for the preparation of nondynamic imine or aldehyde systems for high-throughput screening, although this would not utilize the advantages for in situ screening that a system provides.

## Conclusion

We have developed the first example of *trans*-symmetric reversible exchange in dynamic systems. The results showed that reversible transamination and transimination reactions could be carried out orthogonally or connected to provide access to catalyst-controlled dynamic systems with high level of complexities from simple starting materials. New conditions for aldimine transaminations were also developed, with which quinuclidine was found to be an effective and mild catalyst for the transformation. The TATI system furthermore proved compatible with a wide range of different functional groups, which were well tolerated under the reaction conditions.

The generated dynamic systems could potentially be used for in situ dynamic screening or as starting point systems for combinatorial multicomponent reactions. Efforts to expand the scope of TATI systems for metal sensing and double dynamic materials are currently underway.

## Experimental Section

### General TATI procedure

ZnBr_2_ (2.8 mg, 0.0125 mmol) was added to a dry 2 mL screw-cap vial with activated 3 Å MS (ground, 10 mg) and the mixture was left under N_2_ for 1 h. Benzylic imine (0.25 mmol) was dissolved in anhydrous MeCN (0.25 mL), and **C1** (5.6 mg, 0.05 mmol) was added. The resulting mixture was transferred to the vial and amine was added. The solution was stirred under N_2_ at 50 °C in a sand bath. Reaction monitoring was performed by removing an aliquot (10 μL) of the reaction mixture, filtering through a pad of cotton, and diluting with CDCl_3_, after which an NMR spectrum was recorded.

### One-pot protocol for imine condensation–TATI

4-Trifluoromethylbenzaldehyde (66.8 μL, 87.1 mg, 0.5 mmol) was dissolved in anhydrous MeCN (0.5 mL) in a dry 2 mL screw-cap vial with activated 3 Å MS (ground, 100 mg), and benzylamine (81.9 μL, 80.4 mg, 0.75 mmol) was added. The mixture was stirred at RT under N_2_ for 24 h, after which time **C1** (11.1 mg, 0.1 mmol) and ZnBr_2_ (5.6 mg, 0.025 mmol) were added in one batch. The system was subsequently generated in about 24 h, and analyzed as described in the general procedure.

### General procedure for imine synthesis

Aldehyde (3.0 mmol) was dissolved in anhydrous CH_2_Cl_2_ (30 mL) in a dry round-bottomed flask in the presence of activated 4 Å MS (whole beads, ca. 3.0 g). Amine (3.0 mmol) was added dropwise under N_2_, and the solution was stirred slowly under N_2_. The reaction was monitored by ^1^H NMR spectroscopy sampling, and upon consumption of starting materials the reaction mixture was filtered through a pad of Celite and concentrated in vacuo to directly obtain the imine in a typical purity of 98–99 %.

### Isolation protocol

A dynamic imine system was equilibrated from compound **25** (88.3 mg, 0.25 mmol) and 3,4,5-trimethoxybenzylamine (4.3 μL, 4.9 mg, 0.025 mmol) for 48 h under the conditions described in the general procedure. After equilibrium was reached, the reaction solution was diluted with diethyl ether (2 mL) and the organic phase was washed with a saturated aqueous NH_4_Cl solution (2×1 mL) and brine (1 mL). The colorless solution was dried with MgSO_4_, filtered, and concentrated to afford a clean mixture of the four imines (79.1 mg, 90 % mass recovery). No amine or quinuclidine was present in the sample according to NMR spectroscopy analysis, and hydrolysis of the imines under the workup conditions was less than 1 %.

### Hydrolysis of the imine system

The imine systems could also be hydrolyzed to provide mixtures of aldehydes. The imine mixture (0.25 mmol imine content, either crude or purified according to the procedure outlined above) was dissolved in MeOH (1 mL) and a 1 m aqueous solution of HCl (1 mL) was added dropwise. The resulting solution was stirred at RT under air for 2 h, and the aldehydes were subsequently extracted with diethyl ether (3×2 mL). Drying with MgSO_4_, filtering, and concentration yielded the pure aldehydes without any side products in typically 95–99 % mass balance.

## Supporting Information

As a service to our authors and readers, this journal provides supporting information supplied by the authors. Such materials are peer reviewed and may be re-organized for online delivery, but are not copy-edited or typeset. Technical support issues arising from supporting information (other than missing files) should be addressed to the authors.

miscellaneous_information
