# Supplementary material for: trans-Symmetric Dynamic Covalent Systems: Connected Transamination and Transimination Reactions
Source: Chemistry. 2015 Jun 3;21(27):9776–83. doi: 10.1002/chem.201500520 (PMC4517097; doi:10.1002/chem.201500520)
Supplement: Supplementary file 1 — miscellaneous_information [file chem0021-9776-sd1.pdf]

CHEMISTRY
A European Journal

Supporting Information

trans-Symmetric Dynamic Covalent Systems: Connected Transamination and Transimination Reactions

Fredrik Schaufelberger, Lei Hu, and Olof Ramström*[a]

chem_201500520_sm_miscellaneous_information.pdf

Supporting information

Transsymmetric Dynamic Covalent Systems: Connected Transamination and Transimination Reactions

*Fredrik Schaufelberger, Lei Hu and Olof Ramström**

KTH - Royal Institute of Technology, Department of Chemistry,
Teknikringen 30, S-10044 Stockholm, Sweden; Email: ramstrom@kth.se

Table of Contents

General methods and materials 1
Control experiments 1
Equilibrium two-point entry 2
Lewis acid screening 2
Compound characterization data 2
GC chromatograms 6
NMR spectra of new compounds 8
References 14
NMR spectral data of DCLs 15
Deconvolution methodology 15

General methods and materials

All chemicals were purchased from commercial suppliers with the highest available purity. Chemicals were used as received, except benzylamine and liquid aldehydes, which were distilled under anhydrous conditions at reduced pressure and stored under N_2. The reactions using air or moisture sensitive compounds were carried out with oven-dried glassware under an atmosphere of N_2. Molecular sieves were pre-activated by heating to 600 °C under reduced pressure for 30 minutes. Anhydrous solvents were passed through alumina columns in a Glass Contour solvent dispensing system and stored over molecular sieves, with the exception of acetonitrile which was dried through fractional distillation (atmospheric pressure) over CaH_2 and stored over 3 Å MS. To assure proper NMR analysis of imines, deuterated chloroform was filtrated through a plug of anhydrous K_2CO_3 to remove acidic impurities, followed by drying over activated 4 Å MS under inert atmosphere. Solvents for workup, extractions and flash column chromatography were analytical grade and used as supplied. HRMS was performed at the Institute of Chemistry at University of Tartu, Estonia. ATR-IR spectroscopy was performed on a Thermo Scientific Nicolet iS10 spectrophotometer. GC-FID was performed by a Agilent Technology 6850 Network GC System, using a column (30 m x 0.250 mm x 0.25 μm) containing cyclosil-B as the stationary phase. Reactions were monitored by NMR spectroscopy. A Bruker Avance DMX 500 MHz NMR spectrometer was used for recording ^{1}H-NMR and ^{13}C-NMR, while a Bruker Ascend 400 spectrometer (400 MHz) was used for ^{19}F NMR spectra. Chemical shifts are reported as δ values (ppm) relative to tetramethylsilane (Me_4Si) with residual undeuterated $CHCl_3$ (^{1}H NMR δ 7.26, ^{13}C NMR δ 77.16) or DMSO (^{1}H NMR δ 2.50, ^{13}C NMR δ 39.52) as internal standards. Shifts for ^{19}F-NMR are reported relative $CFCl_3$. All J values are given in Hertz (Hz). Abbreviations for signal coupling are as follows: s = singlet; d = doublet; t = triplet; q = quartet; m = multiplet, br = broad.

Control experiments

To further investigate the TATI system generation, a series of control experiments were conducted. The reactions were conducted according to the general experimental description, but with selective omission of each reagent to probe their importance for the reaction system. The results are summarized in table S1, and are in line with the overall conclusions from the study.

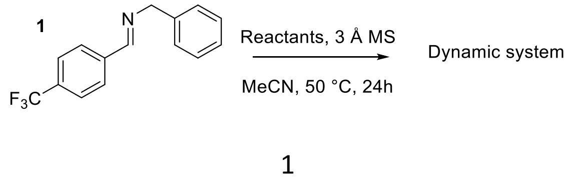

Table S1. Control experiments for TATI DCL generation.

Reactants	Transimination	Transamination
Benzylamine **A1** + $ZnBr_2$ + quinuclidine	Yes	Yes
Benzylamine **A3**+ $ZnBr_2$	Yes	No
Benzylamine **A3**	Yes	No
$ZnBr_2$	No	No
Quinuclidine + $ZnBr_2$	Partial [a]	Yes
Quinuclidine	No	Yes

[a]Low amount of both homoimines detected

Equilibrium two-point entry

To investigate if the coupled dynamic TATI systems were under thermodynamic control, two sets of controlled dual point entry experiments were conducted.

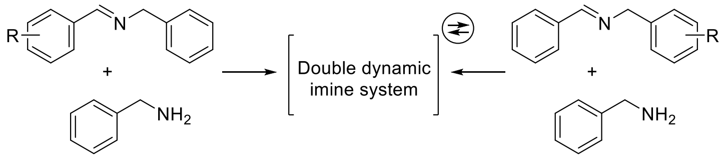

Table S2. Tests for system equilibrium by approach from different starting points.

**Starting imine**	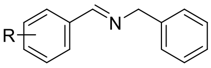	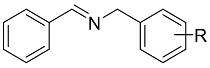	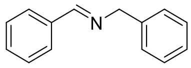	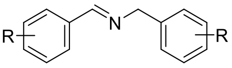
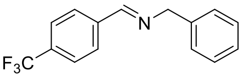 **1**	11%	39%	38%	12%
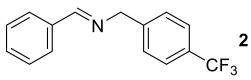 **2**	12%	39%	36%	13%
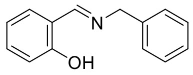 **12**	80%	<1%	13%	6%
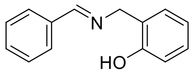 **S3**	70%	2%	16%	12%

Conditions: Imine (0.25 mmol), benzylamine **A1** (0.125 mmol), anhydrous MeCN (0.25 ml), **C1** (0.05 mmol), $ZnBr_2$ (0.0125 mmol), 3 Å MS (10 mg), 50°C, N_2, 48 h.

Lewis acid screening

Aside from $ZnBr_2$, the following Lewis acids were also tested for compatibility with the general TATI conditions: $Sc(OTf)_3$, $Yb(OTf)_3$, $Zn(OTf)_3$, $Zn(OAc)_2$, ZnI_2 and $HgBr_2$. All Lewis acids provided rate accelerations for the equilibration, with only minor discernible differences in equilibrium time. However, $Zn(OAc)_2$ and ZnI_2 led to systems with lower stability with faster degradation and $Sc(OTf)_3$ influenced the equilibrium position significantly, possibly through complexation with the free amine. The choice of $ZnBr_2$ as the catalyst for the system was based on the good solubility of the catalyst, the low toxicity and the excellent long-term stability of the generated dynamic systems.

Compound characterization data

***(E)-N*-benzyl-1-(4-(trifluoromethyl)phenyl)methanimine (1)**[1]

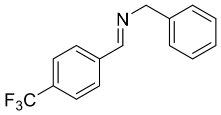

After synthesis according to the general procedure, the compound was further washed with dry Et_2O. White solid. Yield: 89%. **^{1}H NMR** (500 MHz, $CDCl_3$), δ_H = 8.33 (s, 1H), 7.80 (d, *J* = 6.6 Hz, 1H), 7.57 (d, *J* = 6.8 Hz, 1H), 7.30-7.15 (m, 5H), 7.76 (s, 2H); **^{13}C NMR** (125 MHz, $CDCl_3$), δ_c = 160.3, 139.2, 128.8, 132.3 (q, *J* = 32.4 Hz), 128.6, 128.4, 128.0, 127.2, 125.5 (q, *J* = 3.7 Hz), 123.9 (q, *J* = 271 Hz), 65.1; **^{19}F-NMR** (376 MHz, without proton decoupling, $CDCl_3$) δ_F = -62.7 (s); **GC**: flow 2 mL/min, 60 °C for 10 min, 5 °C/min to 200 °C, hold for 12 min, t_R = 39.5 min.

***(E)*-1-phenyl-*N*-(4-(trifluoromethyl)benzyl)methanimine (2)**[2]

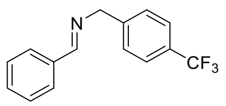

After synthesis according to the general procedure, the compound was further washed with dry Et_2O. The compound had undergone partial transamination during preparation (ca 4%), and as this was the sole impurity the compound was utilized directly in the next step. White solid. Yield: 89%. **^{1}H NMR** (500 MHz, $CDCl_3$), δ_H = 8.44 (s, 1H), 7.82 (dd, J = 7.4, 1.9 Hz, 2H), 7.62 (d, J = 8.0 Hz, 2H), 7.50-7.43 (m, 5H), 4.88 (s, 2H); **^{13}C NMR** (125 MHz, $CDCl_3$), δ_c = 162.6, 143.5, 135.9, 131.0, 129.1 (q, J = 32.3 Hz), 128.7, 128.3, 128.1, 125.4 (q, J = 3.7 Hz) 124.3 (q, J = 272.3 Hz), 64.4; **^{19}F-NMR** (376 MHz, without proton decoupling, $CDCl_3$) δ_F = -62.4 (s); **GC**: flow 2 mL/min, 60 °C for 10 min, 5 °C/min to 200 °C, hold for 12 min, t_R = 40.2 min.

***(E)*-*N*-(4-(trifluoromethyl)benzyl)-1-(4-(trifluoromethyl)phenyl)methanimine (3)**[3]

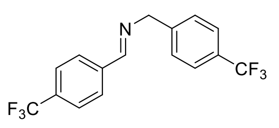

White solid. Yield: 85%. **^{1}H NMR** (500 MHz, $CDCl_3$), δ_H = 8.47 (s, 1H), 7.91 (d, J = 8.0 Hz, 2H), 7.69 (d, J = 8.1 Hz, 2H), 7.62 (d, J = 8.1 Hz, 2H), 7.47 (d, J = 8.0 Hz, 2H), 4.90 (s, 2H); **^{13}C NMR** (125 MHz, $CDCl_3$), δ_c = 161.1, 143.0, 139.0, 132.6 (q, J = 32.3 Hz), 129.4 (q, J = 32.2 Hz), 128.5, 128.1, 125.6 (q, J = 3.7 Hz), 125.5 (q, J = 3.8 Hz), 124.2 (q, J = 270.3 Hz), 123.9 (q, J = 270.7 Hz), 64.4; **^{19}F-NMR** (376 MHz, without proton decoupling, $CDCl_3$) δ_F = -62.4 (s), -62.88 (s); **GC**: flow 2 mL/min, 60 °C for 10 min, 5 °C/min to 200 °C, hold for 12 min, t_R = 40.4 min.

***(E)*-*N*-benzyl-1-phenylmethanimine (4)**[4]

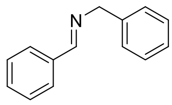

Colorless oil. Yield: 95%. **^{1}H NMR** (500 MHz, $CDCl_3$), δ_H = 8.42 (s, 1H), 7.83-7.81 (m, 2H), 7.46-7.43 (m, 3H), 7.38-7.36 (m, 4H), 7.31-7.27 (m, 1H), 4.86 (s, 2H); **^{13}C NMR** (125 MHz, $CDCl_3$), δ_c = 161.9, 139.3, 136.1, 130.7, 128.6, 128.5, 128.2, 127.9, 126.9, 65.0; **GC**: flow 2 mL/min, 60 °C for 10 min, 5 °C/min to 200 °C, hold for 12 min, t_R = 39.8 min.

***(E)*-*N*-(2-methylbenzyl)-1-(4-(trifluoromethyl)phenyl)methanimine (5)**

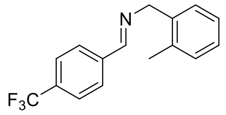

After synthesis according to the general procedure, the compound was further washed with dry Et_2O. White solid. Yield: 96%. **^{1}H NMR** (500 MHz, $CDCl_3$), δ_H = 8.43 (s, 1H), 7.93 (d, J = 8.1 Hz, 2H), 7.72 (d, J = 8.1 Hz, 2H), 7.33-7.30 (m, 1H), 7.27-7.23 (m, 3H), 4.90 (s, 2H), 2.44 (s, 3H); **^{13}C NMR** (125 MHz, $CDCl_3$), δ_c = 160.2, 139.3, 136.9, 136.3, 132.2 (q, J = 32.4 Hz), 130.3, 128.5, 128.4, 127.3, 126.1, 125.5 (q, J = 3.8 Hz), 123.9 (q, J = 270.7 Hz), 62.7, 19.3; **^{19}F-NMR** (376 MHz, without proton decoupling, $CDCl_3$) δ_F = -62.7 (s); **MP** 37-39°C; **HRMS** found 278.1151, calc. for $C_{16}H_{15}F_3N+$ [M+H+] 278.1151; **IR** (ATR, neat).

***(E)*-3-((benzylimino)methyl)benzonitrile (10)**

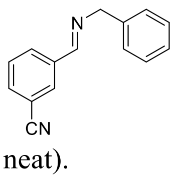

White solid. Yield: 96%. **^{1}H NMR** (500 MHz, $CDCl_3$), δ_H = 8.31 (s, 1H), 8.01 (s, 1H), 7.92 (d, J = 7.9 Hz, 1H), 7.62 (d, J = 7.7 Hz, 1H), 7.45 (t, J = 7.8 Hz, 1H), 7.31-7.25 (m, 4H), 7.23-7.19 (m, 1H), 4.78 (s, 2H); **^{13}C NMR** (125 MHz, $CDCl_3$), δ_c = 159.2, 138.6, 137.2, 133.8, 132.2, 131.7, 129.4, 128.6, 128.0, 127.2, 118.3, 113.0, 64.9; **MP** 31-32°C; **HRMS** found 221.1073, calc. for $C_{15}H_{13}N_2+$ [M+H+] 221.1073; **IR** (ATR, neat).

***(E)*-*N*-benzyl-1-(4-bromophenyl)methanimine (11)**[4]

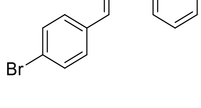

White solid. Yield: 99%. **^{1}H NMR** (500 MHz, $CDCl_3$), δ_H = 8.27 (s, 1H), 7.58 (d, J = 8.4 Hz, 2H), 7.48 (d, J = 8.4 Hz, 2H), 7.307.18 (m, 5H), 4.74 (s, 2H); **^{13}C NMR** (125 MHz, $CDCl_3$), δ_c = 160.6, 139.0, 135.0, 131.8, 129.7, 128.5, 128.0, 127.1, 125.1, 65.0;

***(E)*-2-((benzylimino)methyl)phenol (12)**[4]

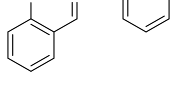

Yellow solid. Yield: 93%. **^{1}H NMR** (500 MHz, $CDCl_3$), δ_H = 13.34 (s, 1H), 8.37 (s, 1H), 7.30-7.17 (m, 7H), 6.89 (d, J = 8.3 Hz, 1H), 6.81 (t, J = 7.4 Hz, 1H), 4.74 (s, 2H); **^{13}C NMR** (125 MHz, $CDCl_3$), δ_c = 165.6, 161.1, 138.1, 132.3, 131.4, 128.7, 127.7, 127.3, 118.8, 118.6, 117.0, 63.2;

***(E)-N*-benzyl-1-(2,4-dichlorophenyl)methanimine (13)**[5]

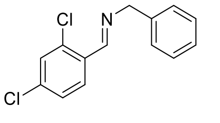

White solid. Yield: >99%. **^{1}H NMR** (500 MHz, $CDCl_3$), δ_H = 8.70 (s, 1H), 7.97 (d, *J* = 8.5 Hz, 1H), 7.33 (d, *J* = 1.9 Hz, 1H), 7.30-7.25 (m, 4H), 7.22-7.17 (m, 2H), 4.78 (s, 2H); **^{13}C NMR** (125 MHz, $CDCl_3$), δ_c = 157.5, 138.8, 137.0, 135.6, 131.7, 129.5, 129.4, 128.6, 128.0, 127.5, 127.1, 65.3;

***(E)-N*-benzyl-1-(4-nitrophenyl)methanimine (14)**[4]

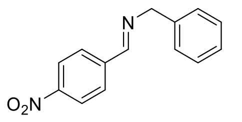

The compound was synthesized according to a procedure by Zarai et al.[6] To a round bottom flask with *p*-nitrobenzaldehyde (3.02 g, 20.0 mmol) in absolute ethanol (40 ml) was dropwise added benzylamine (2.19 ml, 2.14 g, 20.0 mmol) under N_2. The yellow solution was refluxed for 4 h, followed by rapid cooling to 0 °C. The precipitated yellow crystals were collected and dried under high vacuum to afford the pure product (4.14 g, 86%). Yellow crystalline solid. Yield: 86%. **^{1}H NMR** (500 MHz, $CDCl_3$), δ_H = 8.47 (s, 1H), 8.27 (d, *J* = 8.7 Hz, 2H), 7.95 (d, *J* = 8.7 Hz, 2H), 7.40-7.33 (m, 4H), 7.32-7.28 (m, 1H), 4.89 (s, 2H); **^{13}C NMR** (125 MHz, $CDCl_3$), δ_c = 159.4, 149.1, 141.6, 138.4, 128.9, 128.6, 128.0, 127.3, 123.8, 65.2;

***(E)-N*-benzyl-1-(pyridin-4-yl)methanimine (15)**[7]

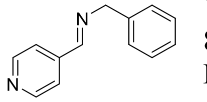

Yellow solid. Yield: 99%. **^{1}H NMR** (500 MHz, $CDCl_3$), δ_H = 8.62 (d, *J* = 5.6 Hz, 2H), 8.30 (s, 1H), 7.56 (d, *J* = 5.6 Hz, 2H), 7.31-7.18 (m, 5H), 4.80 (s, 2H); **^{13}C NMR** (125 MHz, $CDCl_3$), δ_c = 159.9, 150.4, 142.8, 138.4, 128.6, 128.0, 127.3, 122.0, 65.1;

(E)-N-benzyl-1-(pyridin-2-yl)methanimine (16)[4]

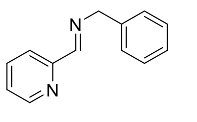

Yellow oil. Yield: 92%. **^{1}H NMR** (500 MHz, $CDCl_3$), δ_H = 8.66 (d, *J* = 4.6 Hz, 1H), 8.49 (s, 1H), 8.07 (d, *J* = 7.9 Hz, 1H), 7.75 (dd, *J* = 11.1, 4.3 Hz, 1H), 7.37-7.27 (m, 6H), 4.89 (s, 2H); **^{13}C NMR** (125 MHz, $CDCl_3$), δ_c = 162.8, 154.6, 149.4, 138.7, 136.5, 128.5, 128.2, 127.1, 124.8, 121.3, 64.9;

***(E)-N*-benzyl-1-(1-methyl-1H-imidazol-2-yl)methanimine (17)**[8]

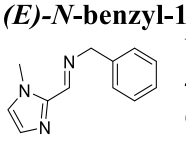

Yellow oil. Yield: 98%. **^{1}H NMR** (500 MHz, $CDCl_3$), δ_H = 8.37 (s, 1H), 7.31-7.26 (m, 4H), 7.23-7.19 (m, 1H), 7.07 (s, 1H), 6.88 (s, 1H), 4.73 (s, 2H), 3.96 (s, 3H); **^{13}C NMR** (125 MHz, $CDCl_3$), δ_c = 154.1, 143.2, 139.2, 129.3, 128.5, 127.8, 127.0, 124.8, 65.4, 35.4;

***(E)-N*-benzyl-1-(furan-2-yl)methanimine (18)**[4]

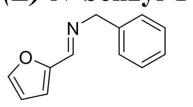

Dark red oil. Yield: 95%. **^{1}H NMR** (500 MHz, $CDCl_3$), δ_H = 8.10 (s, 1H), 7.44 (m, 1H), 7.28-7.24 (m, 4H), 7.20-7.17 (m, 1H), 6.70 (d, *J* = 3.3 Hz, 1H), 6.40 (dd, *J* = 3.3, 1.7 Hz, 1H), 4.72 (s, 2H); **^{13}C NMR** (125 MHz, $CDCl_3$), δ_c = 151.6, 150.3, 144.8, 138.8, 128.5, 128.2, 127.1, 114.1, 111.6, 65.1;

***(E)-N*-benzyl-1-(2-methoxyphenyl)methanimine (19)**[4]

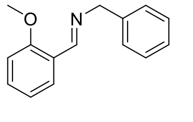

Colorless oil. Yield: 96% . **^{1}H NMR** (500 MHz, $CDCl_3$), δ_H = 8.79 (s, 1H), 7.95 (dd, *J* = 7.7, 1.4 Hz, 1H), 7.33-7.29 (m, 1H), 7.28-7.24 (m, 4H), 7.20-7.16 (m, 1H), 6.91 (t, *J* = 7.5 Hz, 1H), 6.84 (d, *J* = 8.3 Hz, 1H), 4.74 (s, 2H), 3.80 (s, 3H); **^{13}C NMR** (125 MHz, $CDCl_3$), δ_c = 158.8, 158.0, 139.7, 131.9, 128.4, 127.9, 127.5, 126.8, 124.6, 120.8, 110.9, 65.5, 55.5;

***(E)-N*-benzyl-1-(*p*-tolyl)methanimine (20)**[1]

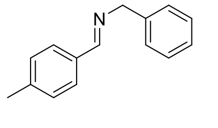

White solid. Yield: 95%. **^{1}H NMR** (500 MHz, $CDCl_3$), δ_H = 8.29 (s, 1H), 7.61 (d, *J* = 7.9 Hz, 2H), 7.29-7.25 (m, 3H), 7.20-7.18 (m, 2H), 7.15 (d, *J* = 7.8 Hz, 2H), 4.74 (s, 2H), 2.32 (s, 3H); **^{13}C NMR** (125 MHz, $CDCl_3$), δ_c = 161.9, 141.0, 139.4, 133.6, 129.3, 128.4, 128.2, 127.9, 126.9, 65.0, 21.5;

***(E)-N*-benzyl-1-(4-fluorophenyl)methanimine (21)**[4]

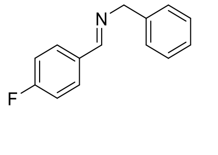

Colorless oil. Yield: 93%. **^{1}H NMR** (500 MHz, $CDCl_3$), δ_H = 8.37 (s, 1H), 7.79 (dd, *J* = 8.4, 5.7 Hz, 2H), 7.38-7.32 (m, 4H), 7.30-7.26 (m, 1H), 7.11 (t, *J* = 8.6 Hz, 1H), 4.82 (s, 2H); **^{13}C NMR** (125 MHz, $CDCl_3$), δ_c = 164.3 (d, *J* = 250.8 Hz), 160.4, 139.2, 132.5 (d, *J* = 3.0 Hz), 130.1 (d, *J* = 8.6 Hz), 128.5, 127.9, 127.0, 115.7 (d, *J* =

21.9 Hz), 64.9; **^{19}F-NMR** (376 MHz, without proton decoupling, $CDCl_3$) δ_F = -109.4 – -109.5 (m).

***(E)-N*-benzyl-1-(naphthalen-2-yl)methanimine** (**22**)[9]

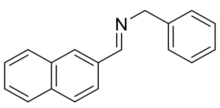

After synthesis according to the general procedure, partial degradation seemed to have occurred. The compound was further recrystallized from hexanes to afford the pure imine. White crystalline solid. Yield: 73%. **^{1}H NMR** (500 MHz, $CDCl_3$), δ_H = 8.47 (s, 1H), 8.01-7.96 (m, 2H), 7.83-7.77 (m, 3H), 7.46-7.41 (m, 2H), 7.32-7.27 (m, 4H), 7.22-7.16 (m, 1H), 4.81 (s, 2H); **^{13}C NMR** (125 MHz, $CDCl_3$), δ_c = 162.0, 139.3, 134.7, 133.8, 133.1, 130.1, 128.6, 128.5, 128.4, 128.0, 127.9, 127.1, 127.0, 126.4, 123.9, 65.1;

***(E)-N*-(4-fluorobenzyl)-1-(4-(trifluoromethyl)phenyl)methanimine (23)**

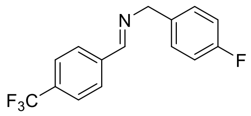

After synthesis according to the general procedure, the compound contained small impurities. The crude mixture was purified by Kugelrohr distillation to afford the pure product. Colorless oil. Yield: 76%. **^{1}H NMR** (500 MHz, $CDCl_3$), δ_H = 8.43 (s, 1H), 7.90 (d, *J* = 8.1 Hz, 2H), 7.68 (d, *J* = 8.2 Hz, 2H), 7.32 (dd, *J* = 8.4, 5.5 Hz, 2H), 7.05 (t, *J* = 8.7 Hz, 2H), 4.83 (s, 2H); **^{13}C NMR** (125 MHz, $CDCl_3$), δ_c = 162.0 (d, *J* = 245.1 Hz), 160.4, 139.1, 134.5 (d, *J* = 3.1 Hz), 132.4 (q, *J* = 32.5 Hz), 129.5 (d, *J* = 8.0 Hz), 128.4, 125.6 (q, *J* = 3.8 Hz), 123.9 (q, *J* = 270.3 Hz), 115.4 (d, *J* = 21.4 Hz), 64.2; **^{19}F-NMR** (376 MHz, without proton decoupling, $CDCl_3$) δ_F = -62.8 (s), -115.7 (m) **HRMS** found 282.0900, calc. for $C_{15}H_{12}F_4N+$ [M+H+] 282.0900; **IR** (ATR, neat).

***(E)-N*-(4-methoxybenzyl)-1-(4-(trifluoromethyl)phenyl)methanimine (24)**

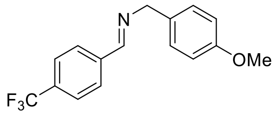

After synthesis according to the general procedure, the compound was further washed with dry Et_2O. White solid. Yield: 99%. **^{1}H NMR** (500 MHz, $CDCl_3$), δ_H = 8.40 (s, 1H), 7.88 (d, *J* = 8.1 Hz, 2H), 7.67 (d, *J* = 8.1 Hz, 2H), 7.26 (d, *J* = 8.6 Hz, 2H), 6.90 (d, *J* = 8.6 Hz, 2H), 4.80 (s, 2H), 3.81 (s, 3H); **^{13}C NMR** (125 MHz, $CDCl_3$), δ_c = 160.0, 158.8, 139.3, 132.2 (q, *J* = 32.3 Hz), 130.8, 129.2, 128.4, 125.5 (q, *J* = 3.8 Hz), 123.9 (q, *J* = 270.7 Hz), 114.0, 64.5, 55.3; **^{19}F-NMR** (376 MHz, without proton decoupling, $CDCl_3$) δ_F = -62.7 (s); **MP** 63-64°C; **HRMS** found 294.1100, calc. for $C_{16}H_{15}F_3NO+$ [M+H+] 294.1100; **IR** (ATR, neat).

***(E)*-1-(4-(trifluoromethyl)phenyl)-*N*-(3,4,5-trimethoxybenzyl)methanimine (25)**

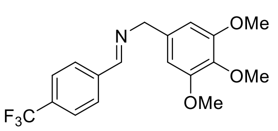

White solid. Yield: >99%. **^{1}H NMR** (400 MHz, $CDCl_3$), δ_H = 8.43 (s, 1H), 7.90 (d, *J* = 8.1 Hz, 2H), 7.68 (d, *J* = 8.2 Hz, 2H), 6.57 (s, 2H), 4.78 (s, 2H), 3.87 (s, 6H), 3.84 (s, 3H); . **^{13}C NMR** (100 MHz, $CDCl_3$), δ_c = 160.4, 153.3, 139.1, 137.0, 134.4, 132.3 (q, *J* = 32.4 Hz), 129.9, 128.5, 125.6 (q, *J* = 3.8 Hz), 123.9 (q, *J* = 270.7 Hz), 104.9, 65.3, 60.8, 56.1; **^{19}F-NMR** (376 MHz, without proton decoupling, $CDCl_3$) δ_F = - 62.8 (s); **MP** 92-94°C; **HRMS** found 354.1313, calc. for $C_{18}H_{19}F_3NO_3+$ [M+H+] 354.1312; **IR** (ATR, neat).

***(E)*-2-((benzylideneamino)methyl)phenol** (**S3**)[10]

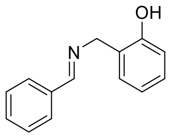

Brown solid. This compound equilibrated rapidly in solution with its ring-closed form (84:16 open/closed form in $CDCl_3$, 97:3 in DMSO-d_6). Yield: 87%. **^{1}H NMR** (500 MHz, DMSO-d_6), δ_H = 9.49 (s, br, 1H), 8.45 (s, 1H), 7.77 (dd, *J* = 7.0, 2.4 Hz, 2H), 7.57 – 7.42 (m, 3H), 7.16 (d, *J* = 6.6 Hz, 1H), 7.09 (dt, *J* = 8.8, 7.1 Hz, 1H), 6.83 (d, *J* = 7.7 Hz, 1H), 6.77 (t, *J* = 7.4 Hz, 1H), 4.72 (s, 2H); **^{13}C NMR** (125 MHz, DMSO-d_6), δ_c = 161.6, 155.0, 136.1, 130.6, 129.2, 128.7, 127.9, 127.8, 125.5, 118.9, 115.0, 58.7;

***(E)*-2-(((2-hydroxybenzyl)imino)methyl)phenol (S4)**[11]

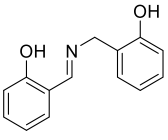

The compound was generated by mixing salicyl aldehyde (61.1 mg, 0.5 mmol) and 2-hydroxybenzylamine (61.6 mg, 0.5 mmol) in a dry round-bottom flask in anhydrous CH_2Cl_2 (8 ml). After stirring at r.t. for 12 h, the product had precipitated out of the reaction. Filtration and subsequent washing with cold CH_2Cl_2 generated the pure product as a yellow solid (64.8 mg, 0.29 mmol). Yield: 57%. **^{1}H NMR** (500 MHz, DMSO-d_6), δ_H = 13.69 (s, br, 1H), 9.61 (s, br, 1H), 8.64 (s, 1H), 7.50-7.41 (m, 1H), 7.35-7.27 (m, 1H), 7.20-7.08 (m, 2H), 6.94-6.74 (m, 4H), 4.73 (s, 2H); **^{13}C NMR** (125 MHz, DMSO-d_6), δ_c = 166.1, 160.9, 155.2, 132.3, 131.6, 129.3, 128.5, 124.4, 119.0, 118.7, 118.4, 116.5, 115.1, 57.0;

***(1S,2R,4S,5R)*-2-(*(R)*-butoxy(6-methoxyquinolin-4-yl)methyl)-5-vinylquinuclidine (C4)**

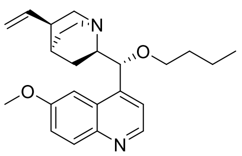

This is a known compound that was synthesized according to the procedure by Shi et al.[12] Yellow oil. Yield: 84%. **^{1}H NMR** (500 MHz, $CDCl_3$), δ_H = 8.74 (d, *J* = 4.4 Hz, 1H), 8.03 (d, *J* = 9.2 Hz, 1H), 7.42 (d, *J* = 4.2 Hz, 1H), 7.36 (dd, *J* = 9.2, 2.6 Hz, 1H), 7.30 (s, 1H), 5.76 – 5.66 (m, 1H), 5.04 (s, 1H), 4.93 (d, *J* = 17.1 Hz, 1H), 4.88 (d, *J* = 10.3 Hz, 1H), 3.93 (s, 3H), 3.43 (s, 1H), 3.36 (t, *J* = 6.3 Hz, 2H), 3.15 – 3.02 (m, 2H), 2.75-2.67 (m, 1H), 2.62-2.58 (m, 1H), 2.25 (s, 1H), 1.81-1.36 (m, 9H), 0.91 (t, *J* = 7.4 Hz, 3H); **^{13}C NMR** (125 MHz, $CDCl_3$), δ_c = 157.7, 147.6, 145.3, 144.7, 142.1, 131.8, 127.4, 121.5, 118.7, 114.1, 101.2, 81.7, 69.3, 60.1, 57.3, 55.7, 43.3, 40.2, 32.2, 28.0, 27.9, 22.1, 19.5, 13.9;

4-(*(R)*-butoxy(*(1S,2R,4S,5R)*-5-vinylquinuclidin-2-yl)methyl)quinolin-6-ol (C5)

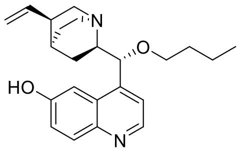

This is a known compound that was synthesized according to the procedure by Shi et al.[12] White solid. Yield: 53%. **^{1}H NMR** (500 MHz, $CDCl_3$), δ_H = 11.44 (s, br, 1H), 8.69 (d, *J* = 4.4 Hz, 1H), 8.28 (s, 1H), 8.00 (d, *J* = 9.0 Hz, 1H), 7.40 (d, *J* = 4.1 Hz, 1H), 7.29 (dd, *J* = 9.0, 1.9 Hz, 1H), 5.68 – 5.55 (m, 1H), 5.36 (s, 1H), 4.91 (d, *J* = 17.1 Hz, 1H), 4.86 (d, *J* = 10.3 Hz, 1H), 3.67-3.60 (m, 1H), 3.33-3.19 (m, 3H), 3.04 – 2.82 (m, 2H), 2.62-2.55 (m, 1H), 2.40-2.33 (m, 1H), 2.10-2.03 (m, 1H), 1.96-1.89 (m, 1H), 1.86-1.82 (m, 1H), 1.66 – 1.34 (m, 6H), 0.90 (t, *J* = 7.3 Hz, 3H); **^{13}C NMR** (125 MHz, $CDCl_3$), δ_c = 156.8, 146.7, 144.6, 143.7, 140.7, 131.0, 128.1, 123.3, 117.9, 114.9, 108.0, 78.5, 69.1, 59.5, 56.3, 43.3, 39.5, 32.2, 27.8, 26.9, 19.5, 13.9.

GC chromatograms

Conditions: Compound **1** or **2** (0.25 mmol), compound **C1** (50 µmol), $ZnBr_2$ (12.5 µmol), benzylamine **A1** (0.025-0.25 mmol) or compound **A2** (0.125 mmol), MeCN (0.25 ml), 50 °C, 48 h.

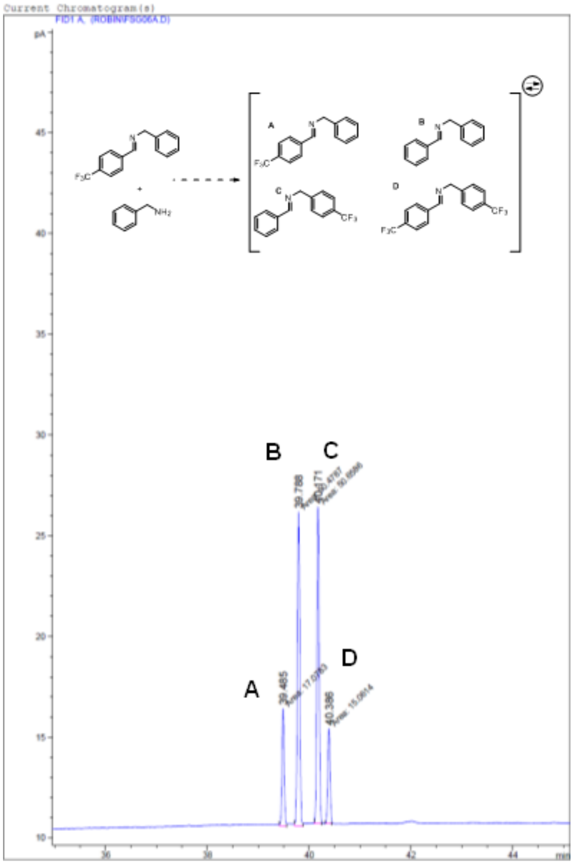

Figure S1. Data for Figure 3b and Figure 4, starting from imine **1**, with amine **A1**.

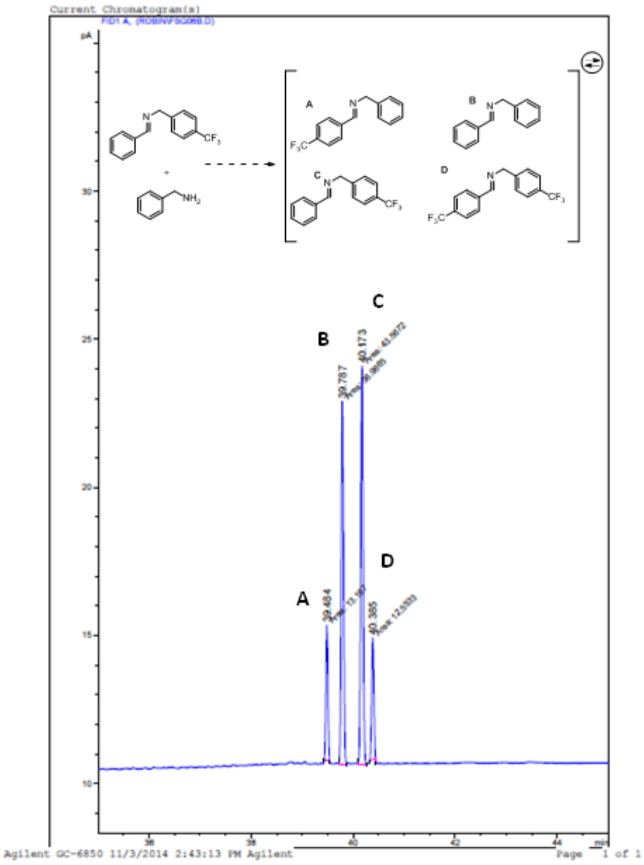

Figure S2. Data for Figure 4, starting from compound **2**, with amine **A1**.

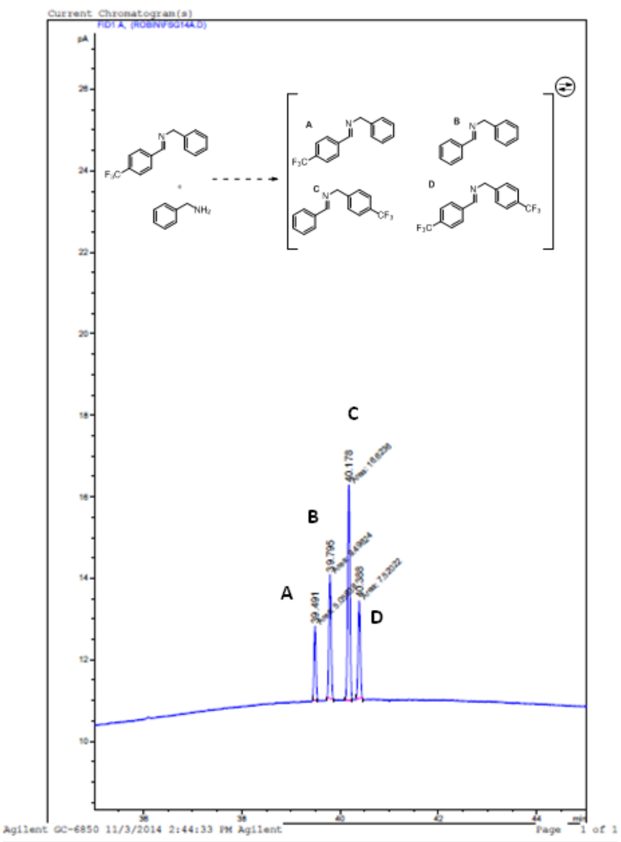

Figure S3. Data for Figure 3a, starting from compound **1,** with amine **A1**.

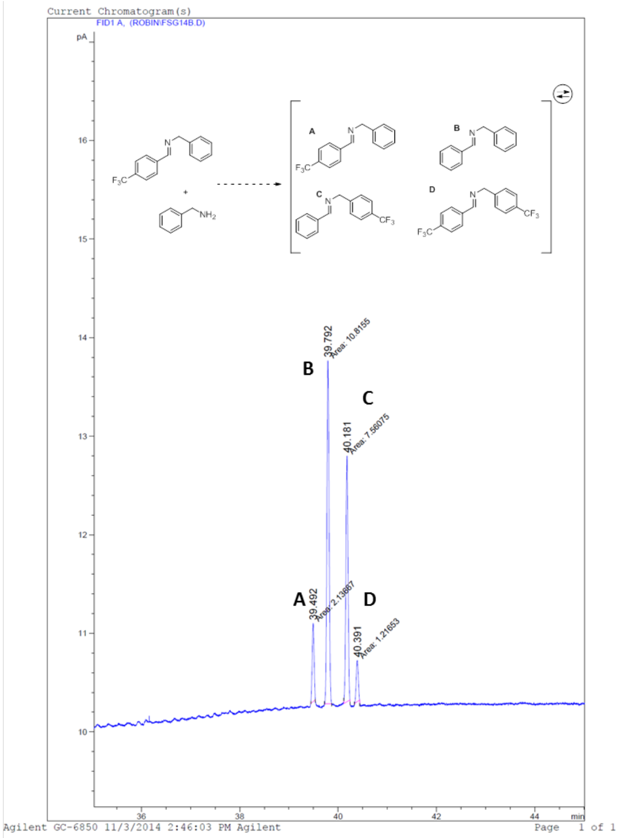

Figure S4. Data for Figure 3c, starting from compound **1,** with amine **A1**.

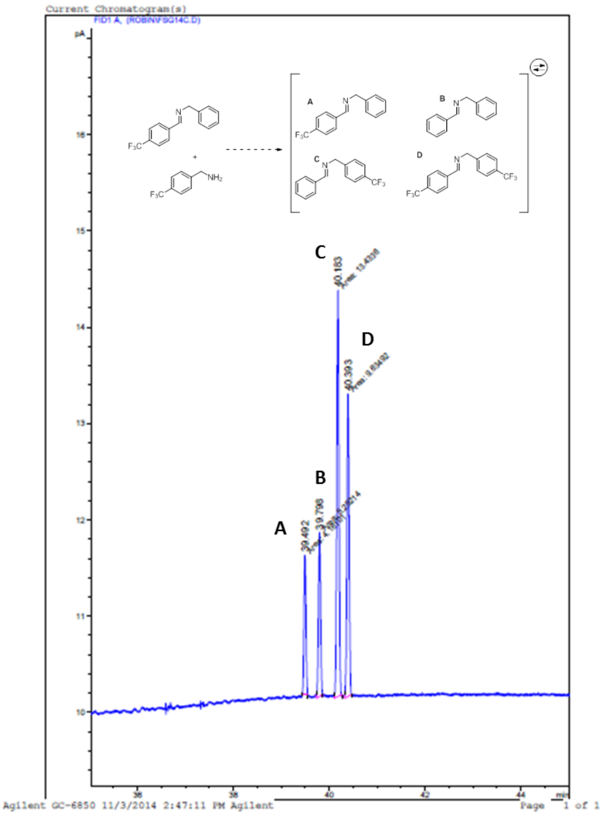

Figure S5. Data for Figure 3d, starting from compound **1**, with amine **A2**.

NMR spectra of new compounds

Compound 5

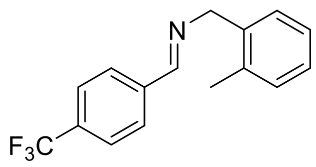

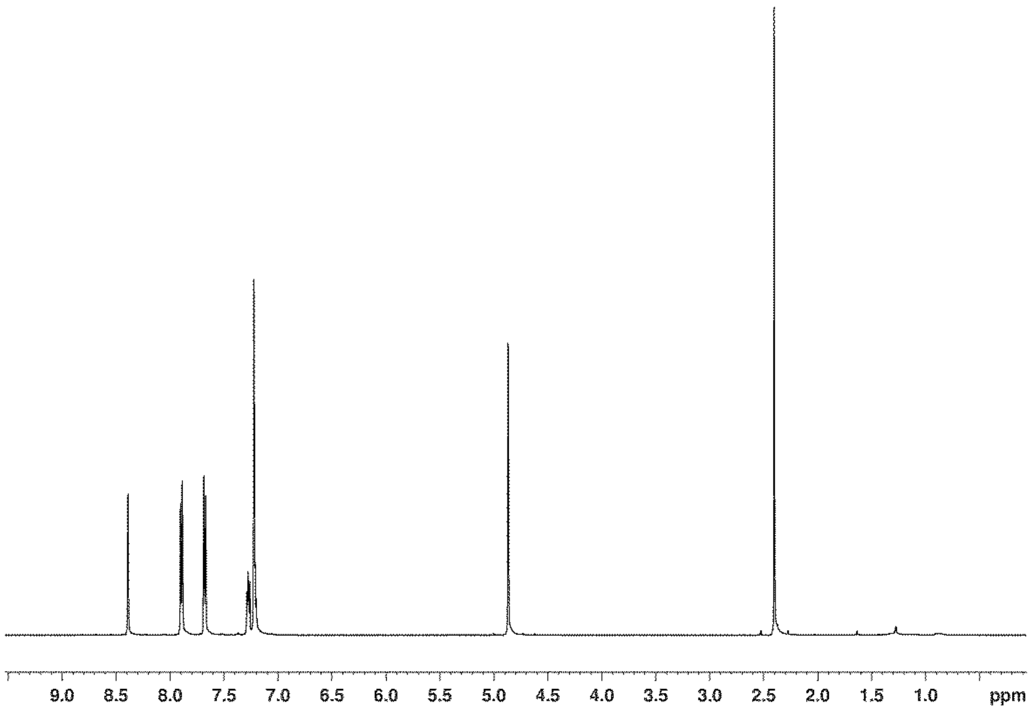

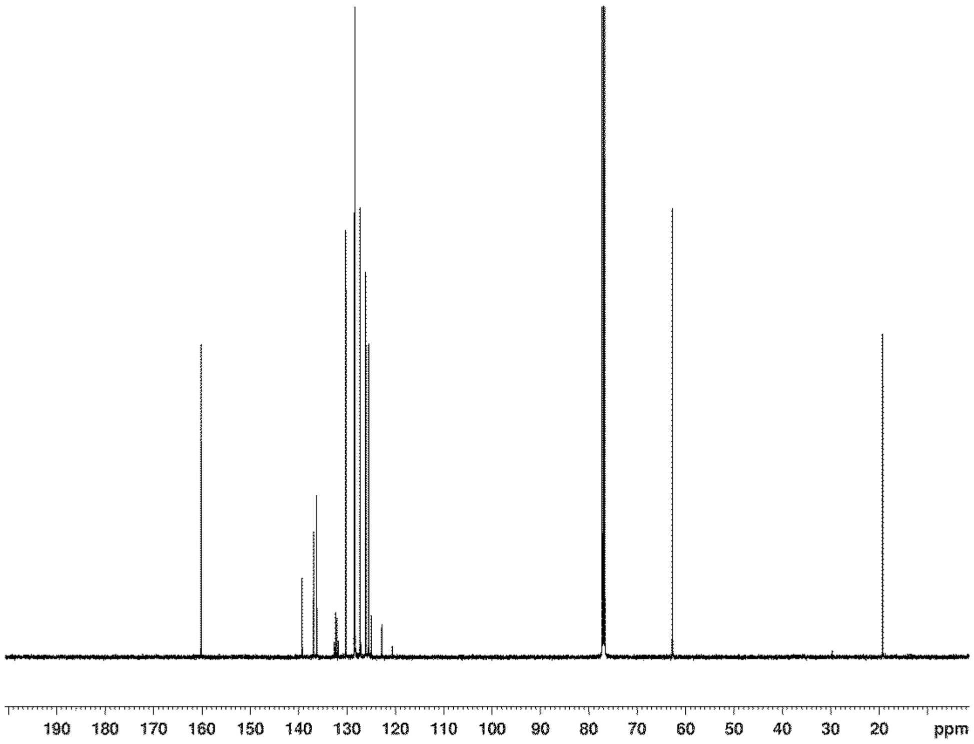

Figure S6. ^{1}H- and ^{13}C-NMR spectra of compound **5**

Compound 10

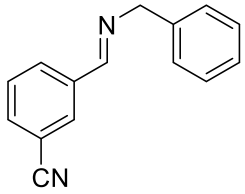

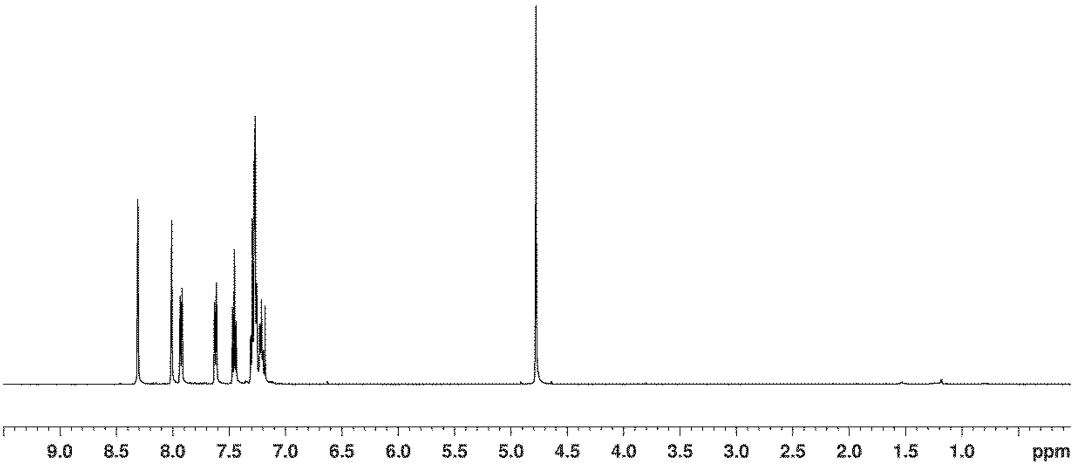

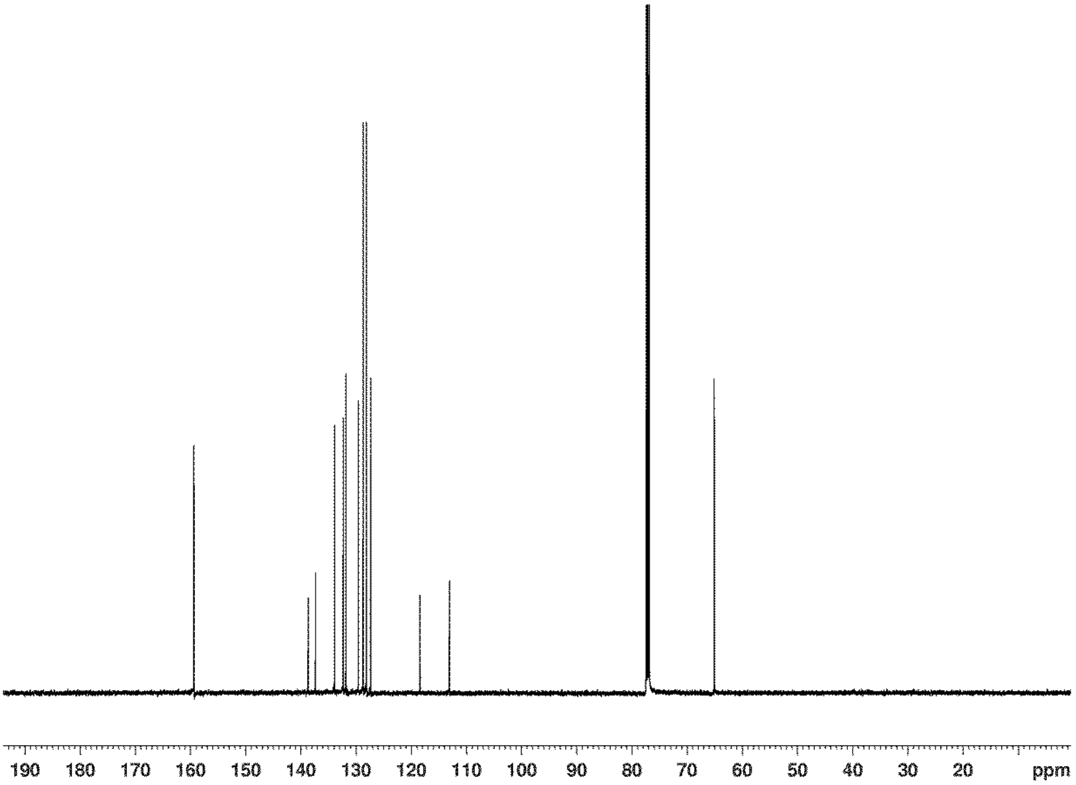

Figure S7. ^{1}H- and ^{13}C-NMR spectra of compound **10**

Compound 23

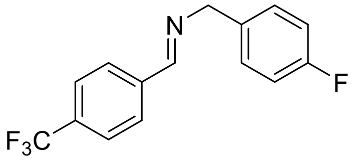

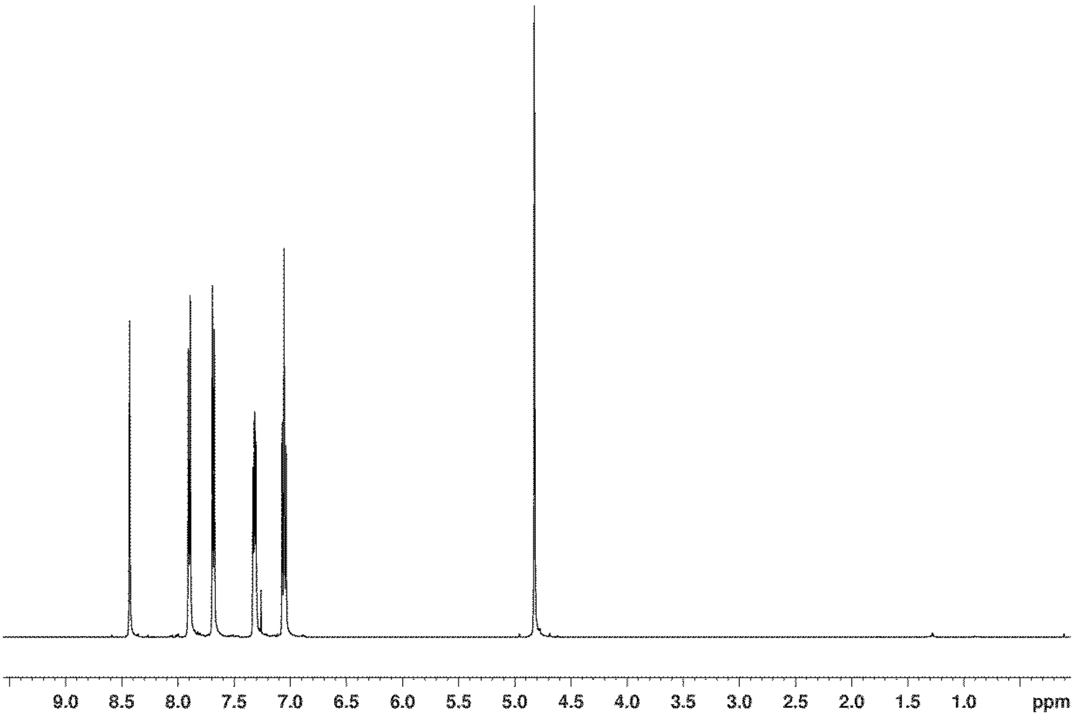

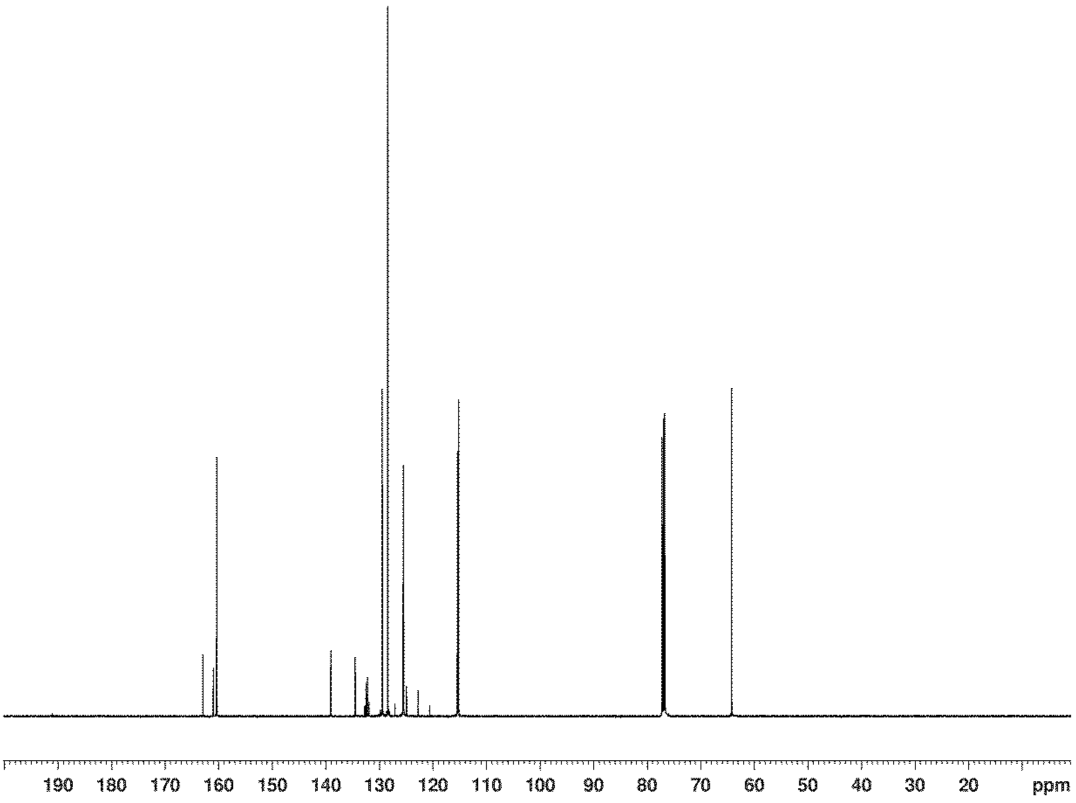

Figure S8. ^{1}H- and ^{13}C-NMR spectra of compound **23**

Compound 24

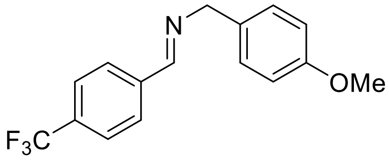

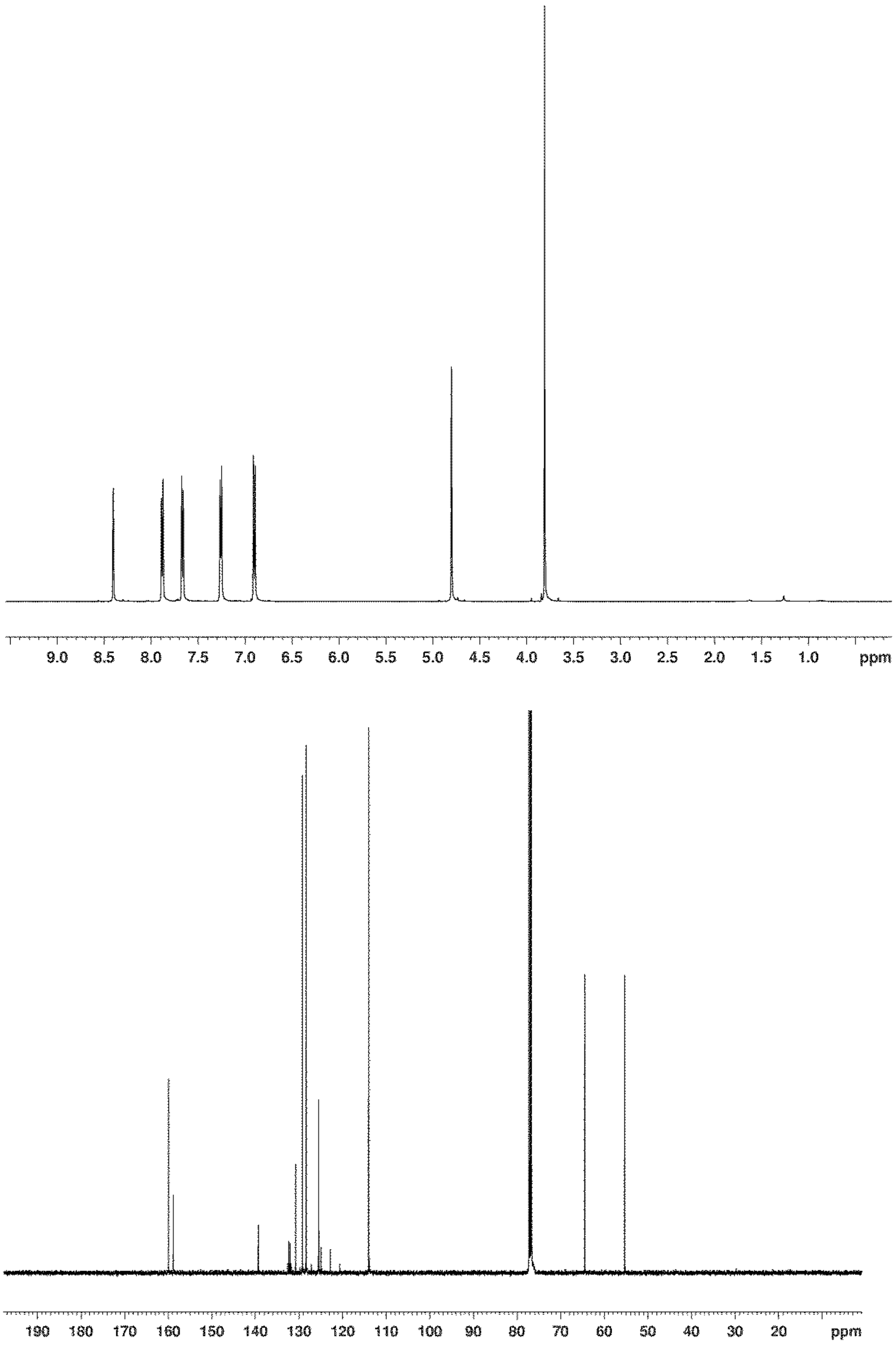

Figure S9. ^{1}H- and ^{13}C-NMR spectra of compound **24**

Compound 25

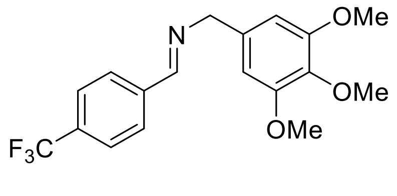

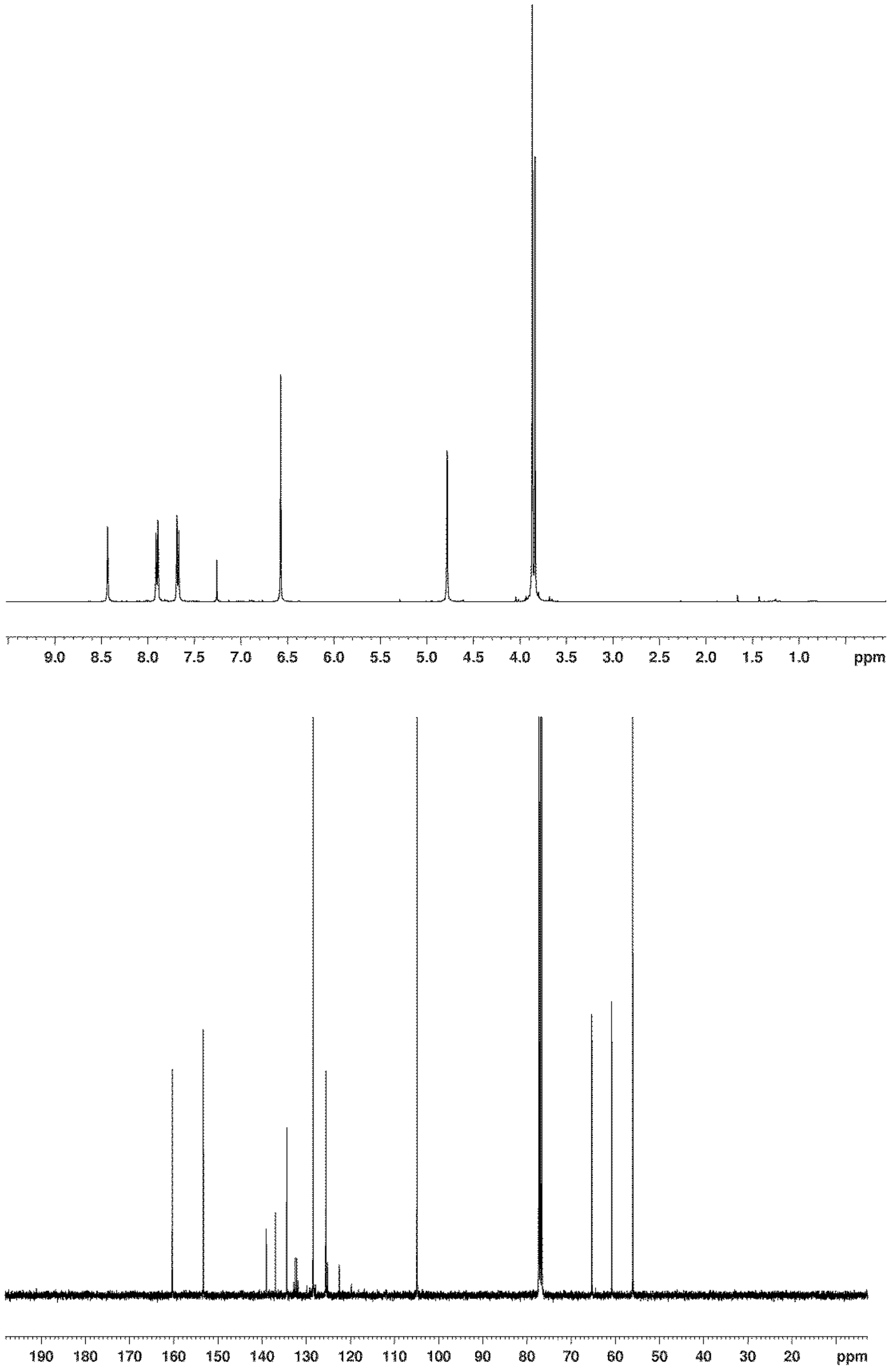

Figure S10. ^{1}H- and ^{13}C-NMR spectra of compound **25**

IR spectra of new compounds

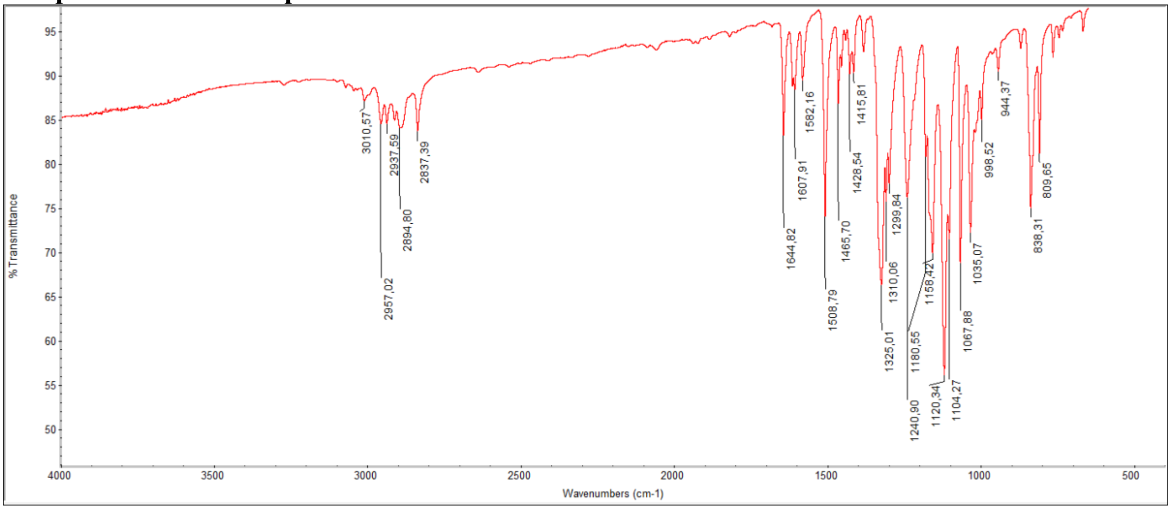

Figure S11. ATR-IR spectrum of compound **5** (ATR corrected, neat)

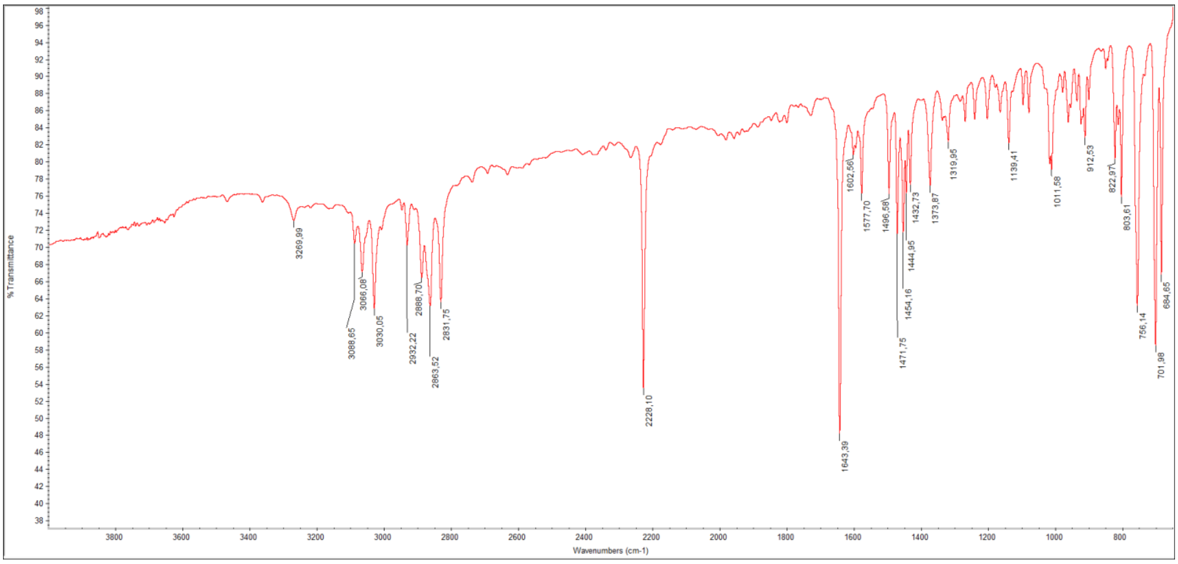

Figure S12. ATR-IR spectrum of compound **10** (ATR corrected, neat)

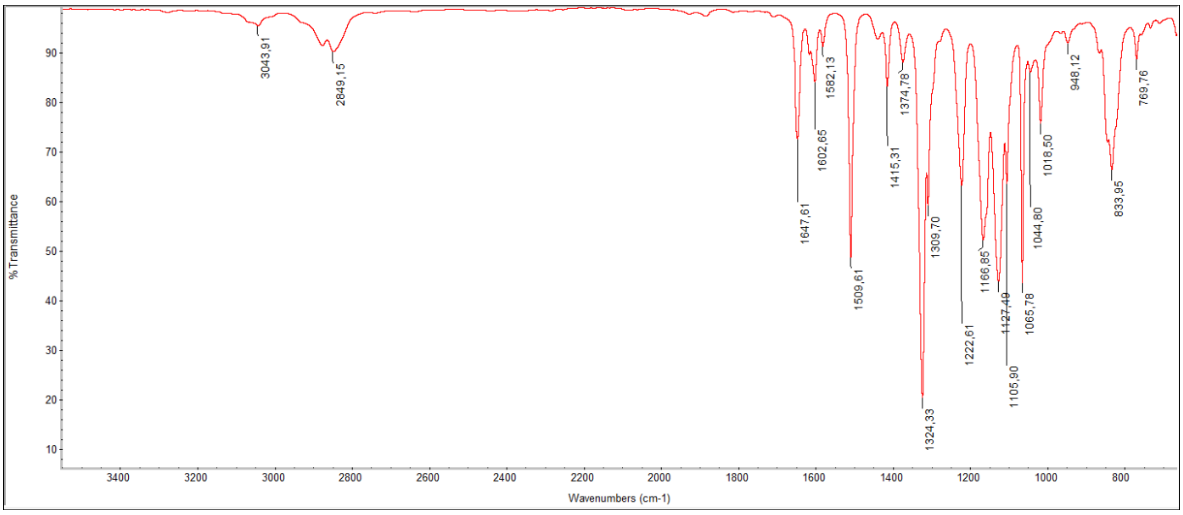

Figure S13. ATR-IR spectrum of compound **23** (ATR corrected, neat)

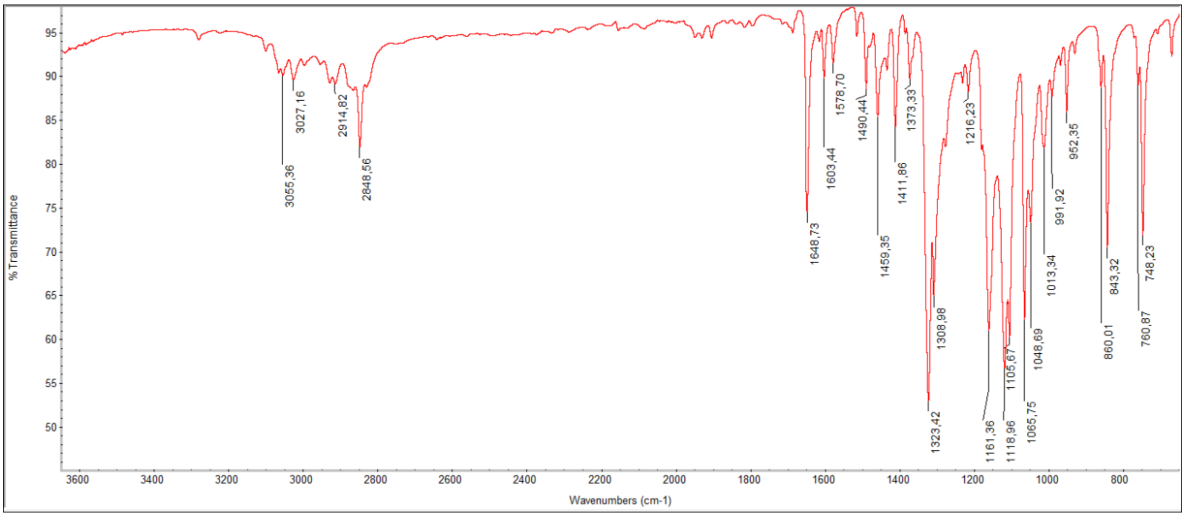

Figure S14. ATR-IR spectrum of compound **24** (ATR corrected, neat)

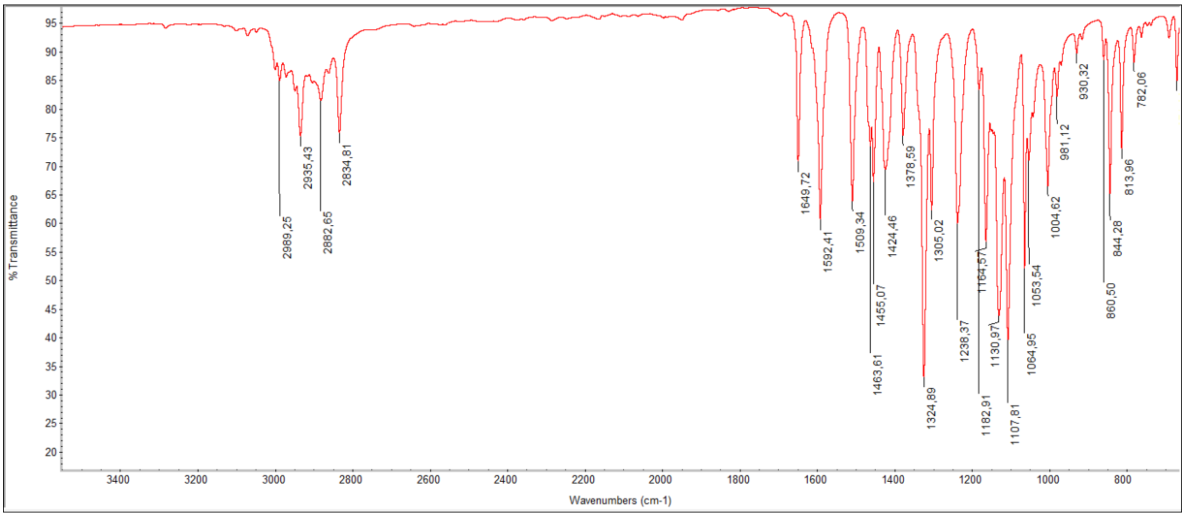

Figure S15. ATR-IR spectrum of compound **25** (ATR corrected, neat)

References

1. Seayad, A. M.; Ramalingam, B.; Yoshinaga, K.; Nagata, T.; Chai, C. L. L. *Org. Lett.* **2010**, *12*, 264–267
2. Pellicciaria, R., Amoria, L.; Kuznetsovab, N.; Zlotskyb, S.; Gioielloa, A. *Tetrahedron Lett*. **2007**. *48*, 4911-4914
3. Wendlandt A. E.; Stahl, S. S. *Org. Lett.* **2012**, *14*, 2850-2853
4. Han L.; Xing, P.; Jiang, B. *Org. Lett.* **2014**, *16*, 3428–3431
5. Crespo, M.; Solans, X.; Font-Bardia, M. *J. Organomet. Chem.* **1996**, *518*, 105-113
6. Jarrahpour, A.; Fadavi, A.; Zarei, M. *Bull. Chem. Soc. Jpn.* **2011**, *84*, 320-327
7. Wilhelms, N.; Kulchat, S.; Lehn, J.-M. *Helv. Chem. Acta* **2012**, *95*, 2635-2651
8. Ferri, N.; Cazzaniga, S.; Mazzarella, L.; Curigliano, G.; Lucchini, G.; Zerla, D.; Gandolfi R.; Facchetti, G.; Pellizzoni, M.; Rimoldi, I. *Bioorg. Med. Chem.* **2013**, *21*, 2379-2386
9. Joly, G. D.; Jacobsen, E. N. *J. Am. Chem. Soc.* **2004**, *126*, 4102-4103
10. Makal, A.; Schilf, W.; Kamienski, B.; Szady-Chelmieniecka, A.; Grech, E.; Wozniak, K. *Dalton Trans.* **2011**, *40*, 421-430
11. Ramesh, K.; Mukherjee, R. *J. Chem. Soc., Dalton Trans.* **1992**, *#1.2*, 83-90
12. Xiao, X.; Xie, Y.; Su, C.; Liu, M.; Shi, Y. *J. Am. Chem. Soc.* **2011**, *133*, 12914-12917

NMR spectral data of DCLs

Deconvolution methodology

Tentative assignment of all imines in the dynamic TATI systems could be made according to the following methodology. Starting imines for each experiment (**A-B** in scheme S2) were fully characterized, as were **B-B** (the two homocombinations **3** and **4**, either one of which were generated in each of the substrate scope experiments). For each entry on the substrate scope list, a quinuclidine-catalyzed transamination according to the optimized conditions developed in table 2 was carried out to identify the direct transamination product **B-A**. Finally, the remaining imine signal **A-A** could be assigned as the unknown homocoupling product. This methodology was verified using the compounds **1** as well as **12**, where all four possible imines were separately synthesized according to the general imine synthesis procedure and characterized independently. In each experiment below, the **B-A** and **A-A** products are separately labeled.

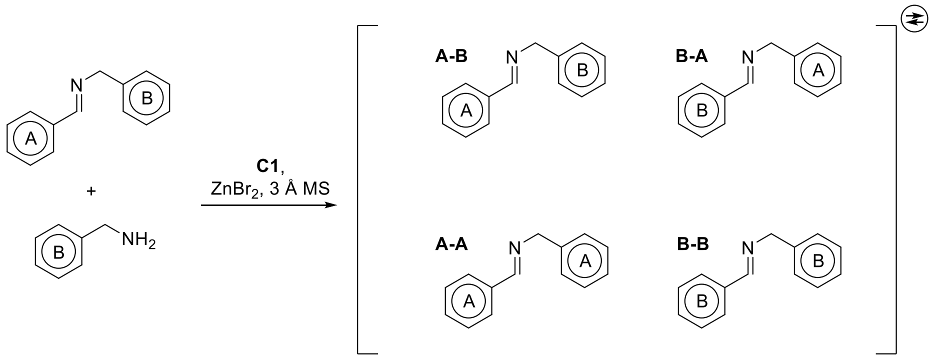

Scheme S2. Deconvolution methodology illustration and nomenclature explanation.

<u>Table 3 entry 1: Compound 1</u>

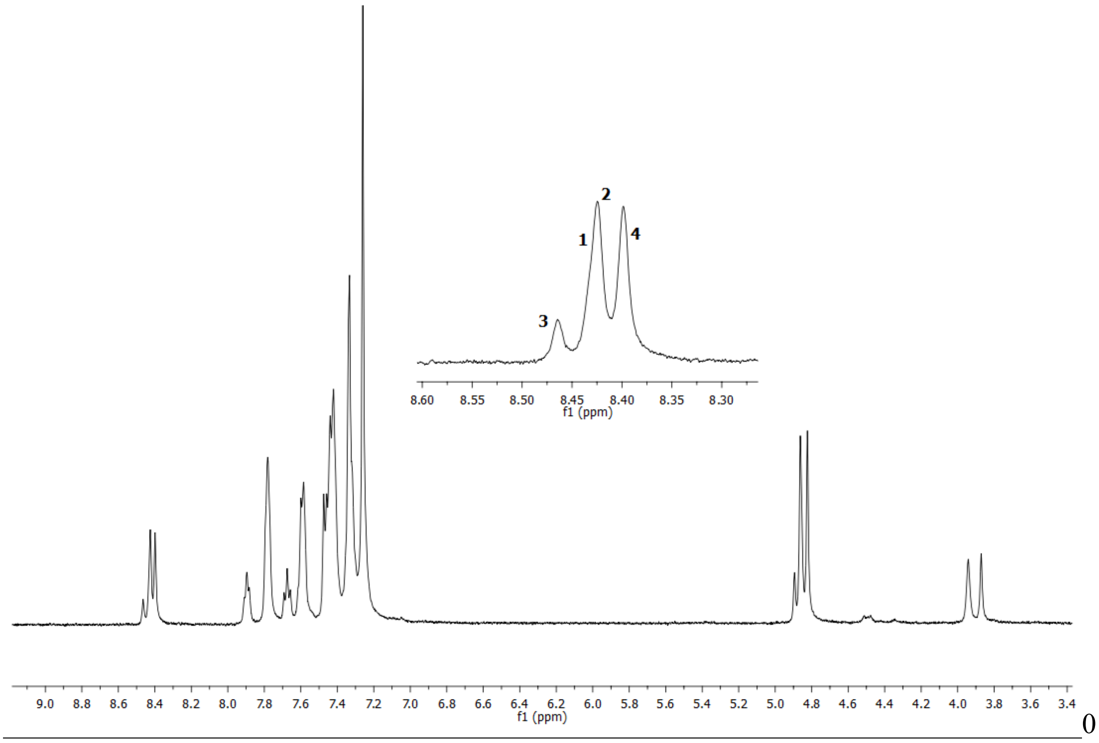

Figure S16. Dynamic system generated from compound **1** according to Table 3.

Table 3 entry 2: Compound 10

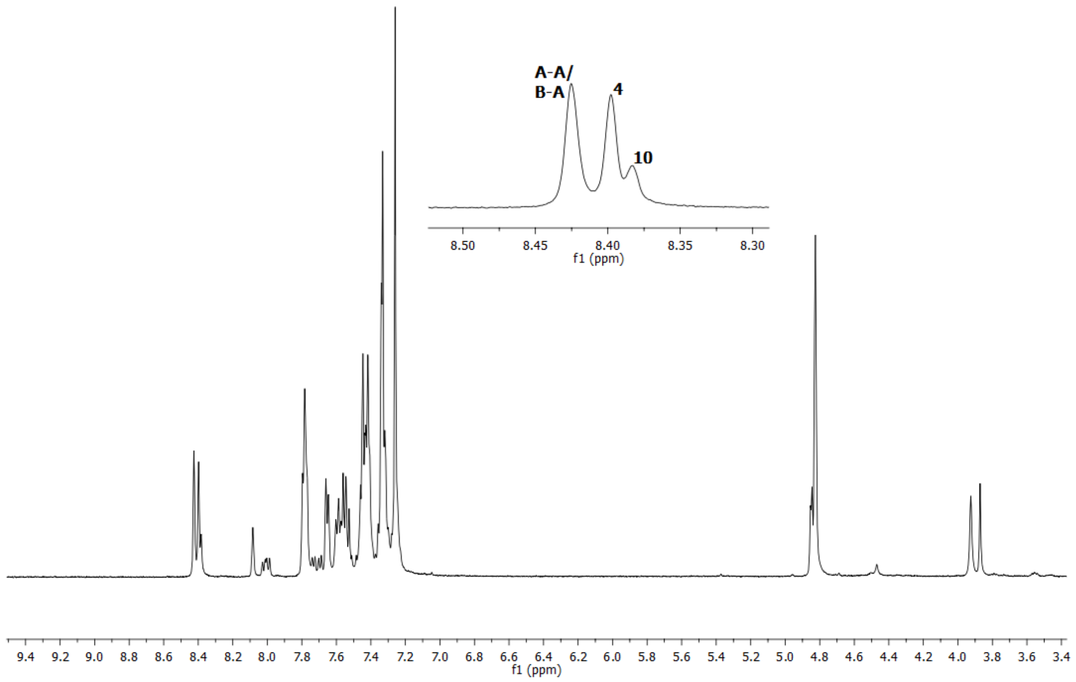

Figure S17. Dynamic system generated from compound **10** according to Table 3. The **A-A** and **B-A** products overlap completely and could not be separated in any tested deuterated solvent.

Table 3 entry 3: Compound 11

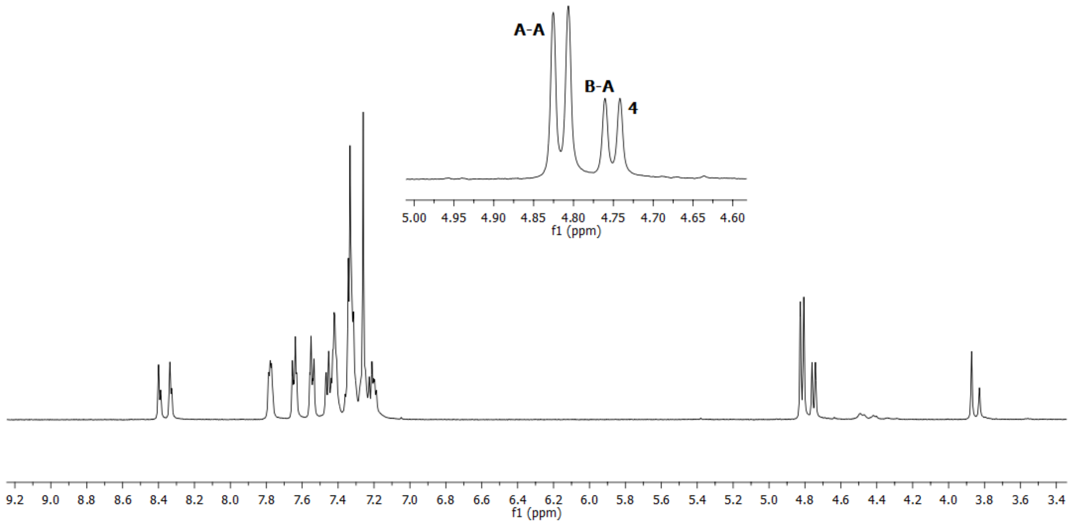

Figure S18. Dynamic system generated from compound **11** according to Table 3.

Table 3 entry 4: Compound 12

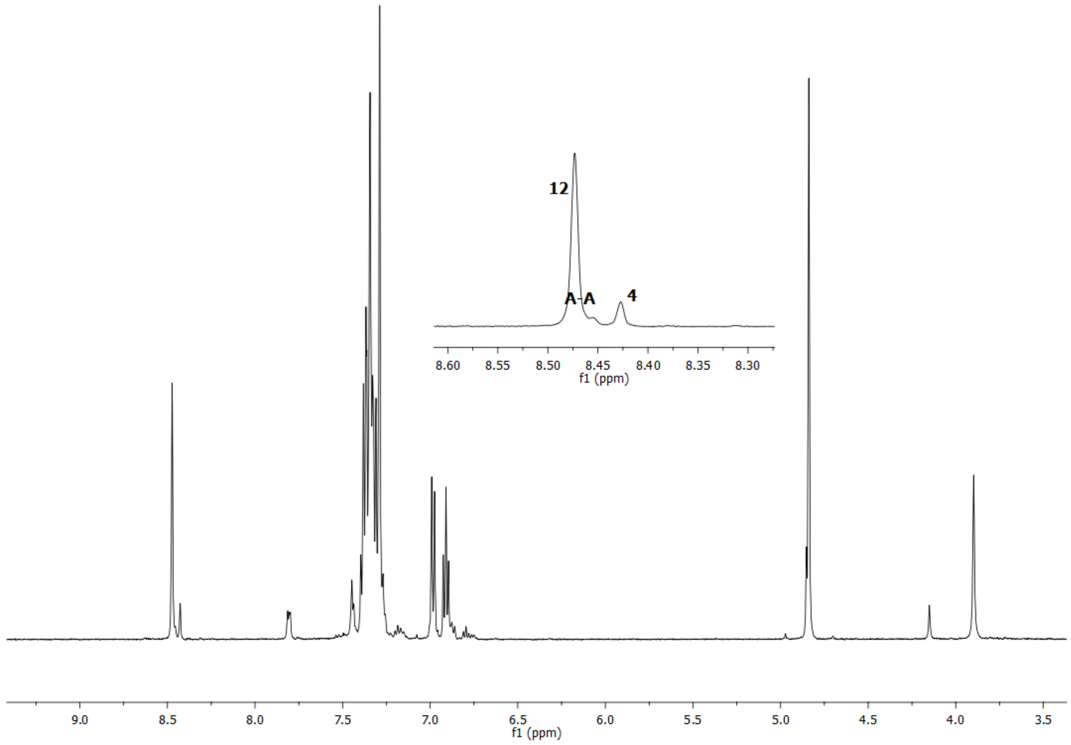

Figure S19. Dynamic system generated from compound **12** according to Table 3. The **B-A** product is not expressed in the system.

Table 3 entry 5: Compound 13

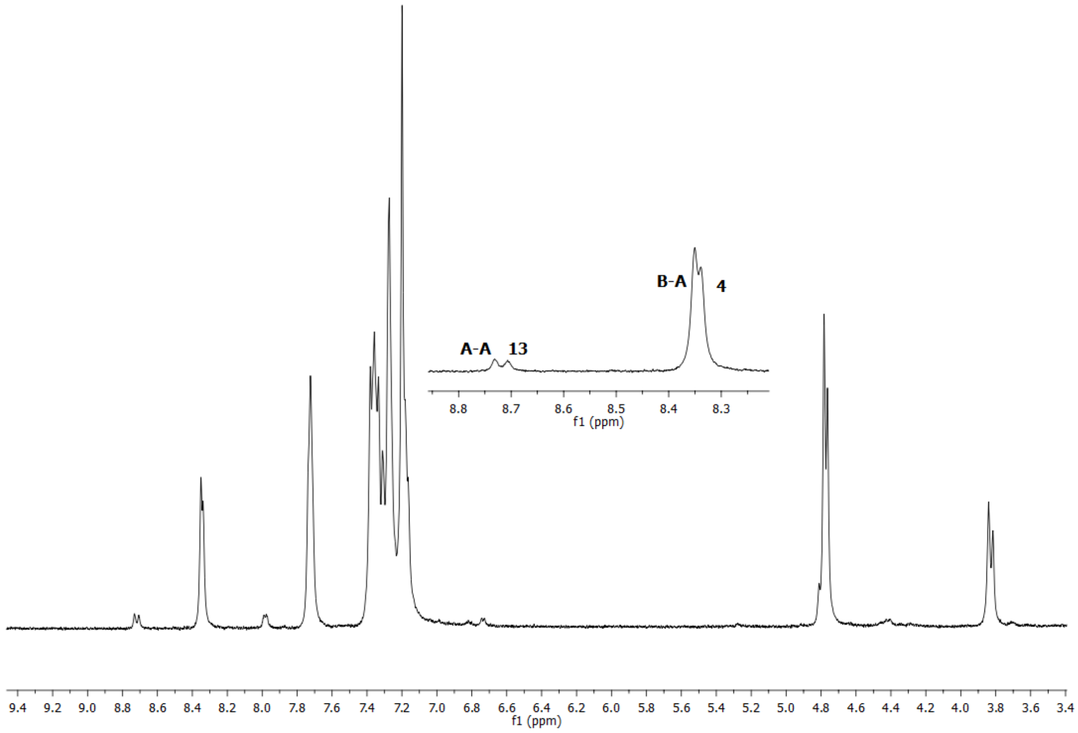

Figure S20. Dynamic system generated from compound **13** according to Table 3.

Table 3 entry 7: Compound 14

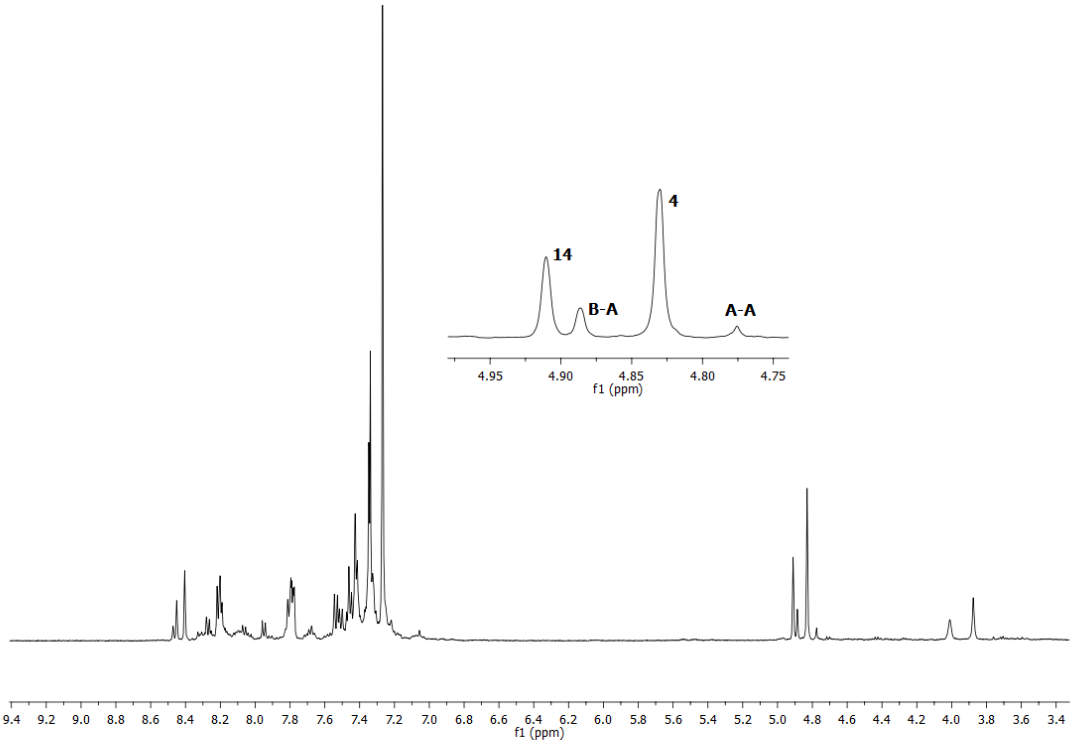

Figure S21. Dynamic system generated from compound **14** according to Table 3.

Table 3 entry 8: Compound 15

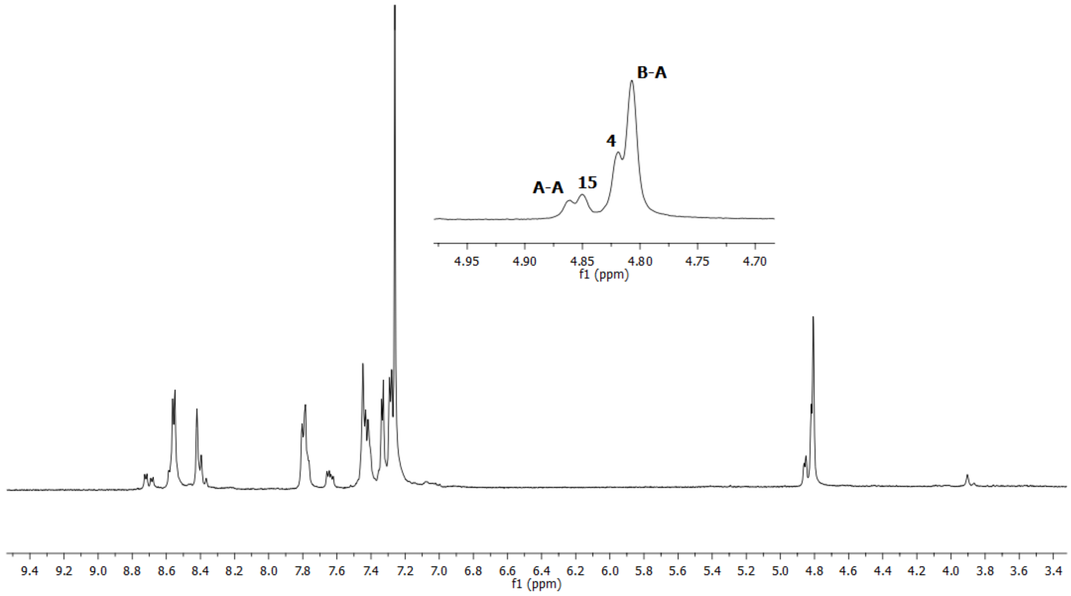

Figure S22. Dynamic system generated from compound **15** according to Table 3.

Table 3 entry 9: Compound 16

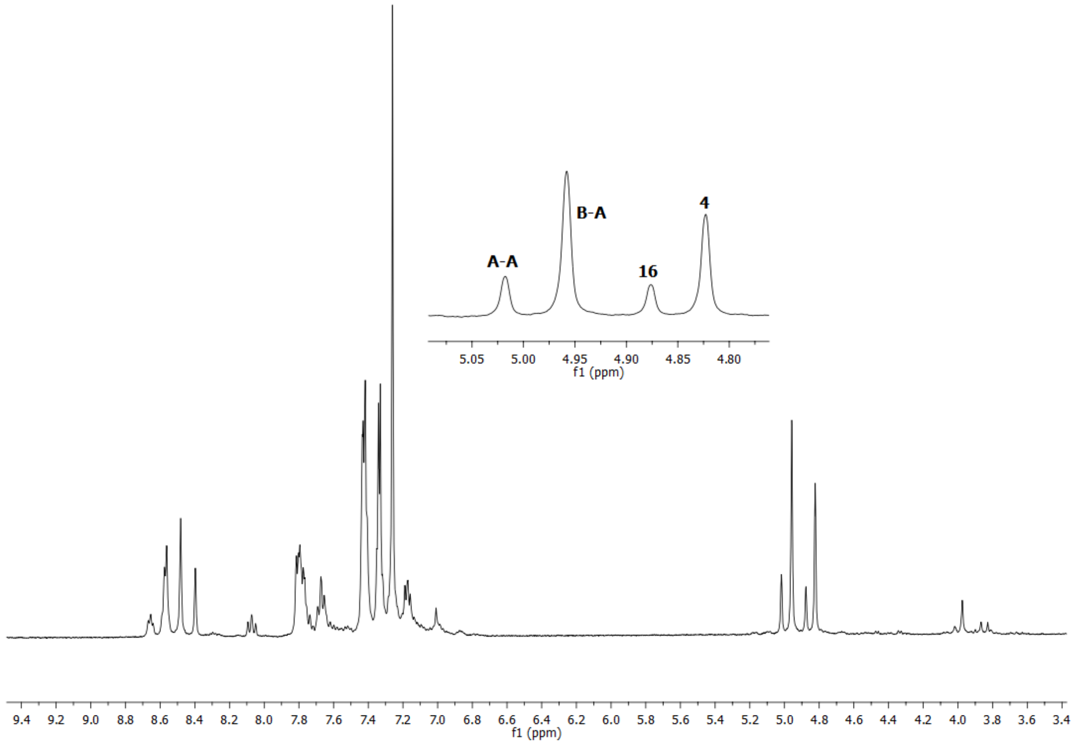

Figure S23. Dynamic system generated from compound **16** according to Table 3.

Table 3 entry 10: Compound 17

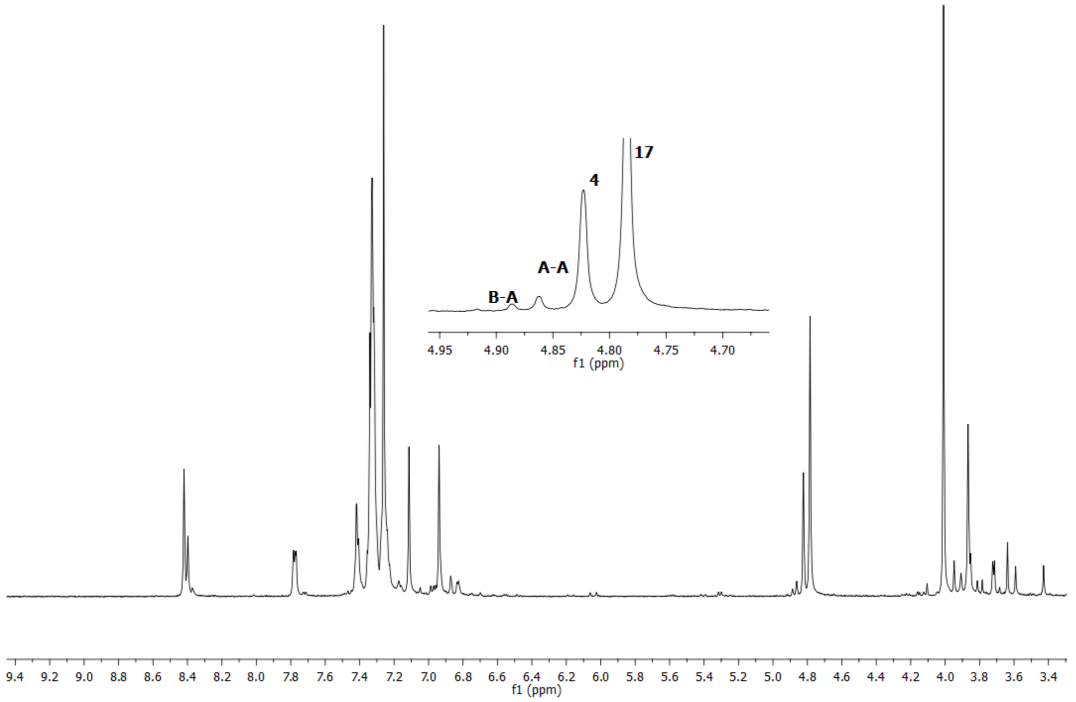

Figure S24. Dynamic system generated from compound **17** according to Table 3.

Table 3 entry 11: Compound 18

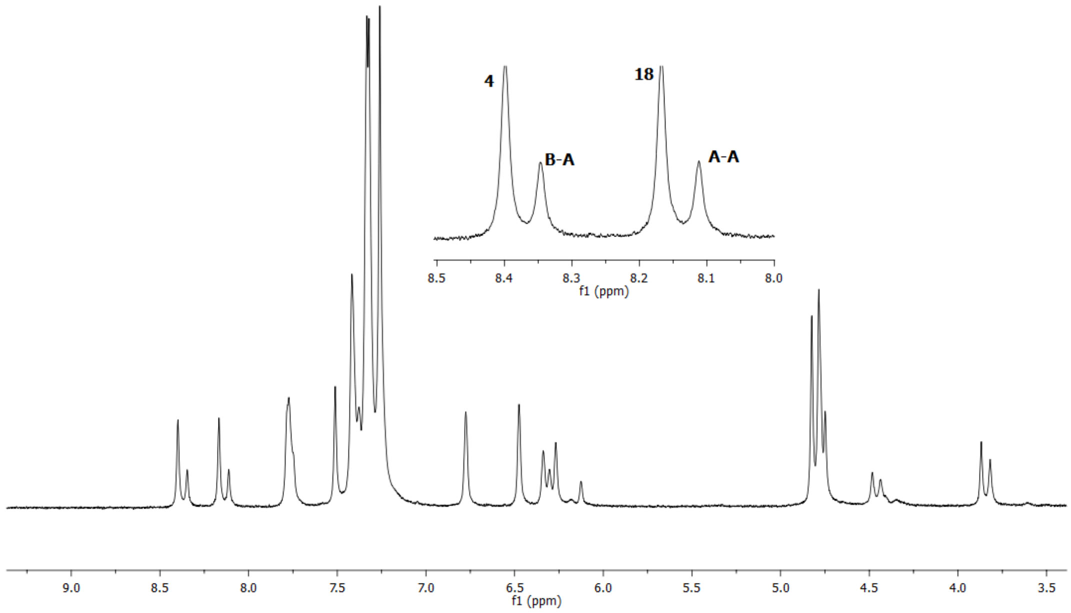

Figure S25. Dynamic system generated from compound **18** according to Table 3.

Table 3 entry 15: Compound 22

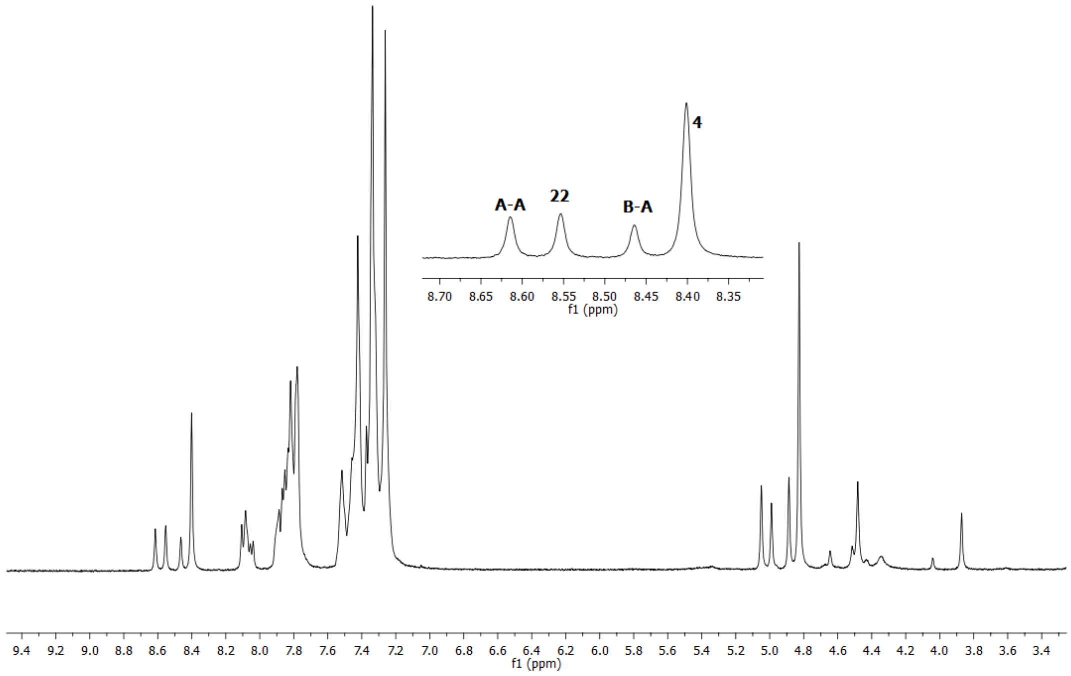

Figure S26. Dynamic system generated from compound **22** according to Table 3.

Table 3 entry 16: Compound 23

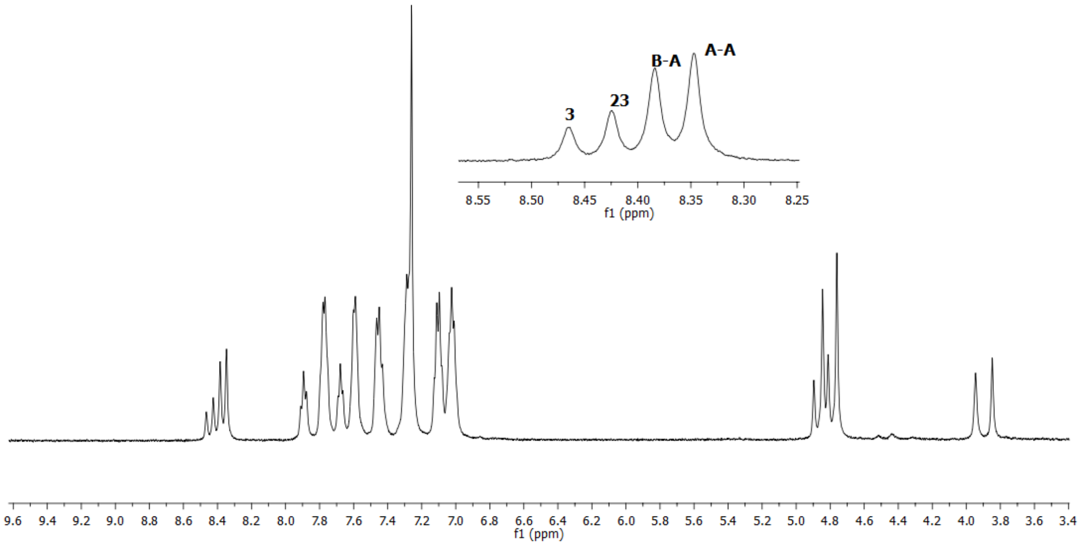

Figure S27. Dynamic system generated from compound **23** according to Table 3.

Table 3 entry 17: Compound 24

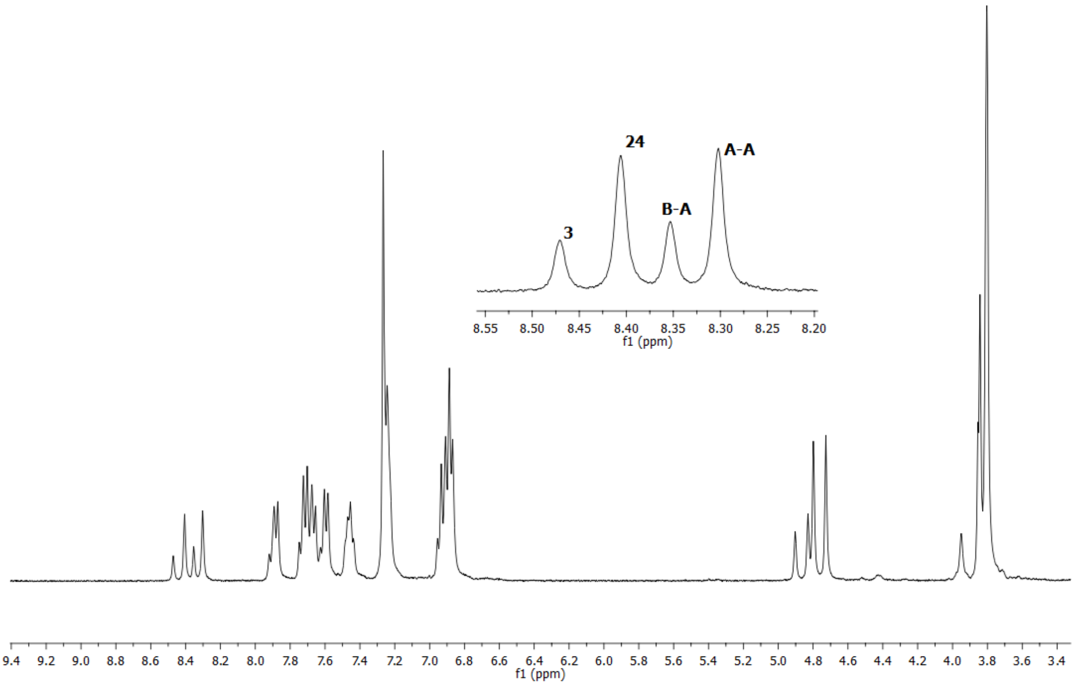

Figure S28. Dynamic system generated from compound **24** according to Table 3.

Table 3 entry 18: Compound 25

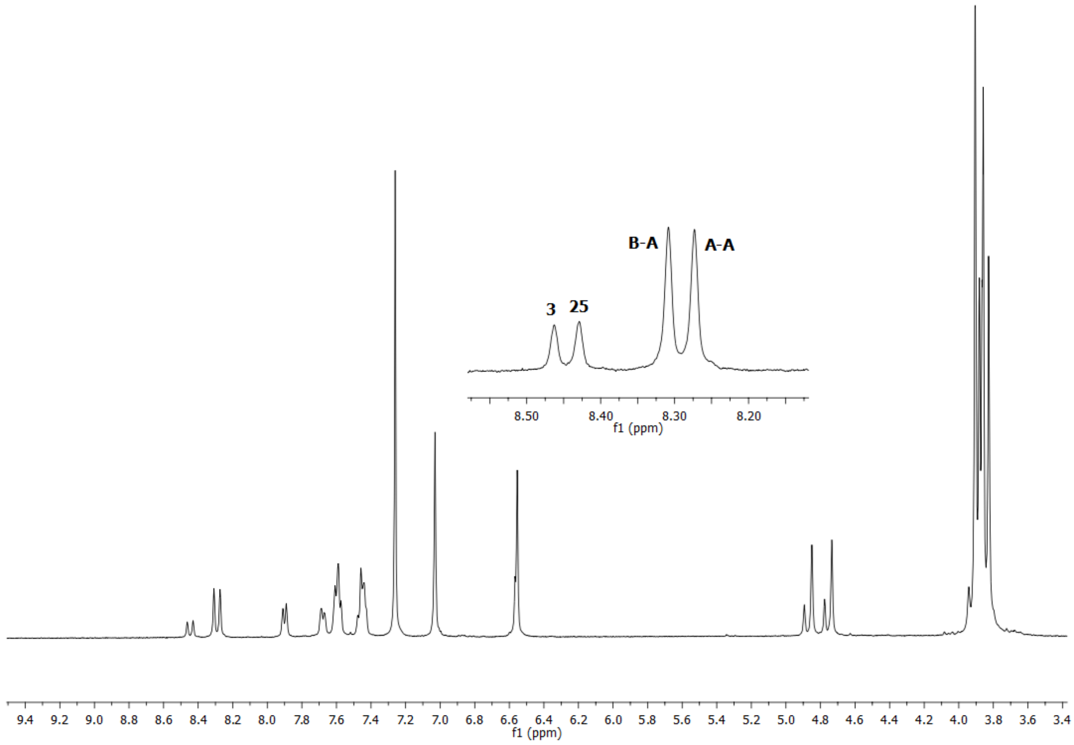

Figure S29. Dynamic system generated from compound **25** according to Table 3.

Table 3 entry 19: Compound 5

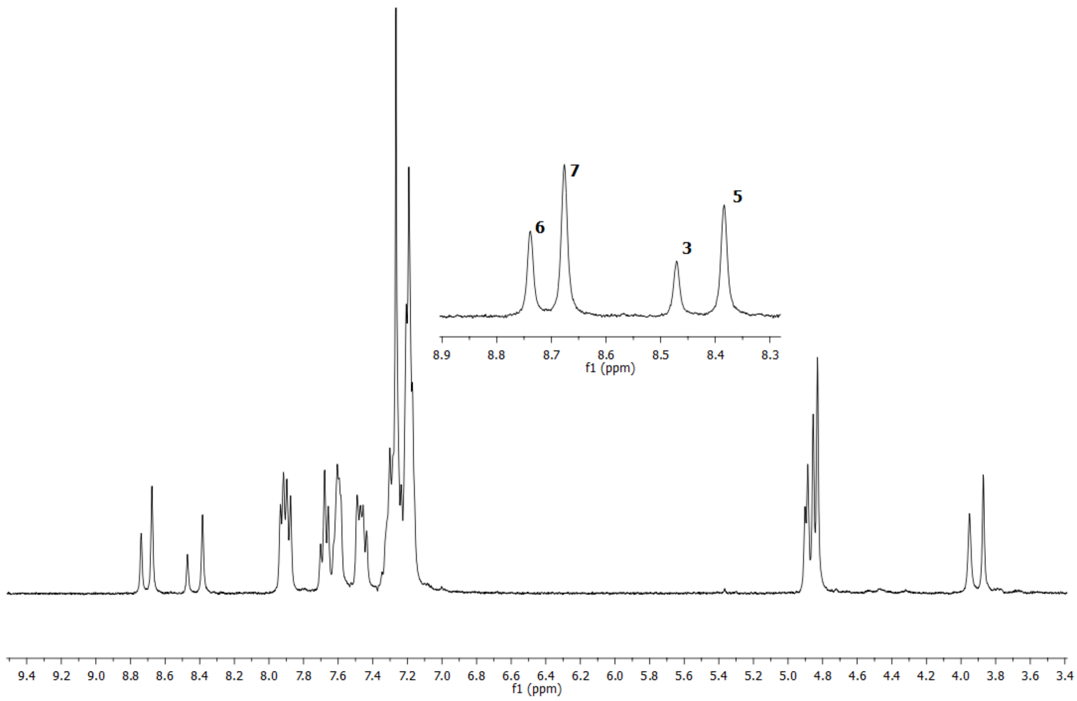

Figure S30. Dynamic system generated from compound **5** according to Table 3.
